# Supplementary material for: Hot spot prediction in protein-protein interactions by an ensemble system
Source: BMC Syst Biol. 2018 Dec 31;12(Suppl 9):132. doi: 10.1186/s12918-018-0665-8 (PMC6311905; doi:10.1186/s12918-018-0665-8)
Supplement: Supplementary file 2 — All relevant MATLAB code. A simple Matlab implement of our predictor. (ZIP 19,742 kb) [file 12918_2018_665_MOESM2_ESM.zip › Additional file 2/Read ASA/ASA website/dbMPIKT/2NZ9A.html]

 NetSurfP - Protein Surface Accessibility and Secondary Structure Predictions

  

|  |  |  |
| --- | --- | --- |
|  |  | NetSurfP - Protein Surface Accessibility and Secondary Structure Predictions  Technical University of Denmark |

  


---

```
# For publication of results, please cite:
# A generic method for assignment of reliability scores applied to solvent accessibility predictions.
# Bent Petersen, Thomas Nordahl Petersen, Pernille Andersen, Morten Nielsen and Claus Lundegaard
# BMC Structural Biology 2009, 9:51 doi:10.1186/1472-6807-9-51
#
# Column 1: Class assignment - B for buried or E for Exposed - Threshold: 25% exposure, but not based on RSA
# Column 2: Amino acid
# Column 3: Sequence name
# Column 4: Amino acid number
# Column 5: Relative Surface Accessibility - RSA
# Column 6: Absolute Surface Accessibility
# Column 7: Z-fit score for RSA prediction
# Column 8: Probability for Alpha-Helix
# Column 9: Probability for Beta-strand
# Column 10: Probability for Coil
E P  2NZ9A                  1    0.645  91.511  -0.595   0.003   0.003   0.994
B F  2NZ9A                  2    0.268  53.747  -1.526   0.021   0.279   0.699
E V  2NZ9A                  3    0.376  57.714  -0.160   0.022   0.359   0.619
B N  2NZ9A                  4    0.251  36.776  -0.895   0.021   0.279   0.699
E K  2NZ9A                  5    0.579 119.018  -0.710   0.019   0.141   0.840
E Q  2NZ9A                  6    0.423  75.584  -0.890   0.018   0.088   0.893
B F  2NZ9A                  7    0.140  28.178   0.529   0.020   0.205   0.775
E N  2NZ9A                  8    0.365  53.480  -0.226   0.021   0.279   0.699
B Y  2NZ9A                  9    0.215  45.839  -0.073   0.020   0.205   0.775
E K  2NZ9A                 10    0.533 109.556  -1.115   0.018   0.088   0.893
E D  2NZ9A                 11    0.346  49.801  -0.453   0.018   0.019   0.964
E P  2NZ9A                 12    0.365  51.737  -1.331   0.018   0.019   0.964
B V  2NZ9A                 13    0.175  26.851  -0.528   0.018   0.047   0.935
E N  2NZ9A                 14    0.455  66.627  -0.152   0.018   0.019   0.964
E G  2NZ9A                 15    0.498  39.208  -1.120   0.018   0.019   0.964
E V  2NZ9A                 16    0.524  80.554   0.524   0.004   0.138   0.858
B D  2NZ9A                 17    0.147  21.125   0.277   0.004   0.514   0.481
B I  2NZ9A                 18    0.032   5.957   0.705   0.001   0.900   0.099
B A  2NZ9A                 19    0.027   2.931   0.596   0.001   0.959   0.040
B Y  2NZ9A                 20    0.044   9.381   0.513   0.001   0.959   0.040
B I  2NZ9A                 21    0.022   4.033   0.954   0.001   0.959   0.040
B K  2NZ9A                 22    0.168  34.558   0.555   0.002   0.816   0.182
B I  2NZ9A                 23    0.062  11.544  -0.374   0.005   0.336   0.660
E P  2NZ9A                 24    0.292  41.392  -0.146   0.019   0.141   0.840
E N  2NZ9A                 25    0.425  62.147  -1.020   0.019   0.141   0.840
E V  2NZ9A                 26    0.363  55.732  -1.129   0.018   0.047   0.935
E G  2NZ9A                 27    0.596  46.897  -2.582   0.018   0.019   0.964
E Q  2NZ9A                 28    0.366  65.332  -1.628   0.053   0.043   0.903
E M  2NZ9A                 29    0.381  76.318  -1.408   0.052   0.084   0.864
E Q  2NZ9A                 30    0.435  77.637   0.366   0.056   0.142   0.802
B P  2NZ9A                 31    0.235  33.346  -0.096   0.069   0.386   0.545
B V  2NZ9A                 32    0.106  16.277   0.403   0.079   0.592   0.329
E K  2NZ9A                 33    0.254  52.166   0.528   0.074   0.484   0.442
B A  2NZ9A                 34    0.050   5.499  -0.579   0.079   0.592   0.329
B F  2NZ9A                 35    0.066  13.206  -0.071   0.085   0.775   0.140
E K  2NZ9A                 36    0.336  69.136   1.223   0.021   0.756   0.223
B I  2NZ9A                 37    0.060  11.156  -0.286   0.022   0.552   0.426
B H  2NZ9A                 38    0.155  28.213   0.456   0.020   0.205   0.775
E N  2NZ9A                 39    0.532  77.943  -0.201   0.018   0.047   0.935
B K  2NZ9A                 40    0.223  45.809  -0.231   0.019   0.141   0.840
B I  2NZ9A                 41    0.028   5.254   0.418   0.021   0.756   0.223
B W  2NZ9A                 42    0.054  13.035   0.149   0.011   0.918   0.071
B V  2NZ9A                 43    0.032   4.918  -0.048   0.011   0.918   0.071
B I  2NZ9A                 44    0.026   4.791   1.279   0.021   0.756   0.223
B P  2NZ9A                 45    0.047   6.712   0.323   0.021   0.451   0.528
B E  2NZ9A                 46    0.170  29.699   0.012   0.020   0.205   0.775
B R  2NZ9A                 47    0.184  42.205  -0.417   0.021   0.279   0.699
B D  2NZ9A                 48    0.169  24.310  -0.293   0.066   0.296   0.638
E T  2NZ9A                 49    0.329  45.688  -0.836   0.066   0.296   0.638
B F  2NZ9A                 50    0.253  50.697  -1.501   0.056   0.142   0.802
E T  2NZ9A                 51    0.475  65.827  -2.066   0.053   0.043   0.903
E N  2NZ9A                 52    0.542  79.363  -0.804   0.053   0.043   0.903
E P  2NZ9A                 53    0.475  67.459  -0.482   0.053   0.043   0.903
E E  2NZ9A                 54    0.602 105.117  -1.676   0.018   0.019   0.964
E E  2NZ9A                 55    0.463  80.956  -1.559   0.018   0.019   0.964
E G  2NZ9A                 56    0.334  26.286  -2.686   0.018   0.019   0.964
E D  2NZ9A                 57    0.524  75.537  -1.025   0.018   0.019   0.964
B L  2NZ9A                 58    0.258  47.313  -2.518   0.018   0.019   0.964
B N  2NZ9A                 59    0.294  42.983  -1.576   0.018   0.019   0.964
E P  2NZ9A                 60    0.538  76.342  -0.394   0.018   0.019   0.964
B P  2NZ9A                 61    0.170  24.052  -2.077   0.058   0.017   0.925
E P  2NZ9A                 62    0.471  66.892  -2.120   0.053   0.043   0.903
E E  2NZ9A                 63    0.497  86.843  -0.822   0.053   0.043   0.903
E A  2NZ9A                 64    0.415  45.777  -1.468   0.052   0.084   0.864
E K  2NZ9A                 65    0.491 101.040  -0.527   0.019   0.141   0.840
E Q  2NZ9A                 66    0.626 111.893  -0.788   0.019   0.141   0.840
E V  2NZ9A                 67    0.330  50.752  -1.088   0.018   0.047   0.935
E P  2NZ9A                 68    0.608  86.275  -1.346   0.018   0.047   0.935
E V  2NZ9A                 69    0.365  56.100  -1.410   0.019   0.141   0.840
E S  2NZ9A                 70    0.326  38.160  -1.581   0.021   0.279   0.699
B Y  2NZ9A                 71    0.263  56.160  -0.545   0.021   0.451   0.528
B Y  2NZ9A                 72    0.242  51.694   0.001   0.022   0.359   0.619
B D  2NZ9A                 73    0.194  27.898   0.219   0.019   0.141   0.840
E S  2NZ9A                 74    0.459  53.842  -1.048   0.018   0.047   0.935
E T  2NZ9A                 75    0.545  75.591  -1.067   0.053   0.043   0.903
B Y  2NZ9A                 76    0.244  52.057   0.382   0.052   0.084   0.864
B L  2NZ9A                 77    0.159  29.186  -0.047   0.052   0.084   0.864
E S  2NZ9A                 78    0.517  60.569  -0.081   0.052   0.084   0.864
E T  2NZ9A                 79    0.509  70.557  -0.188   0.113   0.043   0.844
E D  2NZ9A                 80    0.515  74.154  -0.701   0.181   0.016   0.803
E N  2NZ9A                 81    0.668  97.795  -0.232   0.406   0.004   0.590
E E  2NZ9A                 82    0.611 106.812   0.052   0.694   0.003   0.303
E K  2NZ9A                 83    0.279  57.370   0.294   0.782   0.003   0.216
E D  2NZ9A                 84    0.357  51.473  -0.096   0.782   0.003   0.216
E N  2NZ9A                 85    0.511  74.766   1.648   0.923   0.002   0.076
B Y  2NZ9A                 86    0.070  15.023  -0.031   0.970   0.001   0.030
B L  2NZ9A                 87    0.039   7.196   0.502   0.988   0.000   0.012
E K  2NZ9A                 88    0.400  82.198   1.333   0.988   0.000   0.012
B G  2NZ9A                 89    0.110   8.673   0.159   0.988   0.000   0.012
B V  2NZ9A                 90    0.026   3.919   1.096   0.988   0.000   0.012
B T  2NZ9A                 91    0.050   6.880   0.896   0.988   0.000   0.012
E K  2NZ9A                 92    0.341  70.226   1.715   0.988   0.000   0.012
B L  2NZ9A                 93    0.039   7.104  -0.025   0.970   0.001   0.030
B F  2NZ9A                 94    0.053  10.637   0.587   0.970   0.001   0.030
E E  2NZ9A                 95    0.403  70.334   0.814   0.938   0.007   0.055
E R  2NZ9A                 96    0.306  70.005   0.899   0.802   0.014   0.185
B I  2NZ9A                 97    0.062  11.414   0.122   0.522   0.016   0.462
E Y  2NZ9A                 98    0.439  93.921  -0.512   0.181   0.016   0.803
E S  2NZ9A                 99    0.528  61.928  -1.277   0.109   0.005   0.886
E T  2NZ9A                100    0.453  62.873   0.152   0.246   0.004   0.750
E D  2NZ9A                101    0.439  63.289  -0.696   0.694   0.003   0.303
B L  2NZ9A                102    0.128  23.473  -0.064   0.782   0.003   0.216
B G  2NZ9A                103    0.036   2.825  -2.083   0.923   0.002   0.076
E R  2NZ9A                104    0.389  89.173   1.383   0.970   0.001   0.030
E M  2NZ9A                105    0.360  72.056   1.571   0.970   0.001   0.030
B L  2NZ9A                106    0.033   6.024   0.811   0.970   0.001   0.030
B L  2NZ9A                107    0.039   7.123   0.325   0.988   0.000   0.012
E T  2NZ9A                108    0.409  56.714   0.793   0.970   0.001   0.030
B S  2NZ9A                109    0.208  24.389   1.152   0.970   0.001   0.030
B I  2NZ9A                110    0.032   5.939   0.254   0.923   0.002   0.076
B V  2NZ9A                111    0.116  17.875  -0.229   0.858   0.002   0.139
E R  2NZ9A                112    0.465 106.531  -0.312   0.694   0.003   0.303
B G  2NZ9A                113    0.249  19.588  -1.830   0.257   0.016   0.727
B I  2NZ9A                114    0.135  25.012   0.501   0.115   0.016   0.868
B P  2NZ9A                115    0.211  29.884  -1.289   0.018   0.047   0.935
B F  2NZ9A                116    0.152  30.426  -0.484   0.018   0.047   0.935
B W  2NZ9A                117    0.188  45.142  -0.835   0.018   0.047   0.935
E G  2NZ9A                118    0.344  27.049  -1.943   0.018   0.019   0.964
E G  2NZ9A                119    0.531  41.790  -2.090   0.018   0.019   0.964
E S  2NZ9A                120    0.440  51.521  -1.517   0.053   0.043   0.903
E T  2NZ9A                121    0.446  61.902  -1.247   0.018   0.047   0.935
B I  2NZ9A                122    0.249  46.046   0.066   0.053   0.043   0.903
E D  2NZ9A                123    0.478  68.822  -0.442   0.053   0.043   0.903
E T  2NZ9A                124    0.570  79.017  -1.271   0.113   0.043   0.844
E E  2NZ9A                125    0.420  73.461  -1.961   0.113   0.087   0.800
B L  2NZ9A                126    0.283  51.762   0.200   0.113   0.087   0.800
E K  2NZ9A                127    0.354  72.818  -0.963   0.113   0.087   0.800
E V  2NZ9A                128    0.406  62.464  -1.119   0.056   0.142   0.802
E I  2NZ9A                129    0.355  65.712  -1.666   0.020   0.205   0.775
E D  2NZ9A                130    0.393  56.646  -0.557   0.022   0.359   0.619
B T  2NZ9A                131    0.222  30.791  -0.323   0.004   0.616   0.381
E N  2NZ9A                132    0.354  51.840  -0.520   0.004   0.616   0.381
B C  2NZ9A                133    0.294  41.221  -0.948   0.003   0.718   0.279
B I  2NZ9A                134    0.148  27.343  -0.313   0.004   0.616   0.381
E N  2NZ9A                135    0.377  55.149  -0.402   0.021   0.756   0.223
B V  2NZ9A                136    0.110  16.876   0.368   0.021   0.756   0.223
E I  2NZ9A                137    0.416  76.997  -0.261   0.004   0.616   0.381
B Q  2NZ9A                138    0.236  42.221  -0.589   0.005   0.336   0.660
E P  2NZ9A                139    0.529  75.079  -1.302   0.004   0.085   0.910
E D  2NZ9A                140    0.629  90.668  -1.174   0.018   0.019   0.964
E G  2NZ9A                141    0.514  40.428  -1.660   0.018   0.047   0.935
E S  2NZ9A                142    0.484  56.701  -2.278   0.020   0.205   0.775
E Y  2NZ9A                143    0.454  97.041  -0.644   0.022   0.552   0.426
E R  2NZ9A                144    0.403  92.195  -0.128   0.023   0.655   0.322
E S  2NZ9A                145    0.296  34.750  -0.626   0.023   0.655   0.322
E E  2NZ9A                146    0.355  62.018  -0.226   0.004   0.616   0.381
B E  2NZ9A                147    0.261  45.667  -1.088   0.005   0.336   0.660
B L  2NZ9A                148    0.087  15.930  -0.182   0.005   0.262   0.733
B N  2NZ9A                149    0.076  11.141  -1.071   0.004   0.616   0.381
B L  2NZ9A                150    0.023   4.156   1.086   0.001   0.900   0.099
B V  2NZ9A                151    0.025   3.873   0.725   0.001   0.959   0.040
B I  2NZ9A                152    0.017   3.145   1.057   0.001   0.959   0.040
B I  2NZ9A                153    0.030   5.531  -0.049   0.002   0.816   0.182
B G  2NZ9A                154    0.049   3.825  -0.409   0.005   0.262   0.733
B P  2NZ9A                155    0.169  23.981  -1.007   0.005   0.045   0.951
B S  2NZ9A                156    0.218  25.538  -0.814   0.018   0.047   0.935
B A  2NZ9A                157    0.223  24.564  -1.355   0.018   0.047   0.935
B D  2NZ9A                158    0.260  37.495  -0.793   0.018   0.088   0.893
B I  2NZ9A                159    0.067  12.395  -0.463   0.022   0.359   0.619
B I  2NZ9A                160    0.216  39.904  -0.507   0.022   0.359   0.619
E Q  2NZ9A                161    0.427  76.244   0.185   0.066   0.296   0.638
E F  2NZ9A                162    0.334  67.134  -1.001   0.020   0.205   0.775
E E  2NZ9A                163    0.451  78.842  -0.920   0.022   0.359   0.619
B C  2NZ9A                164    0.115  16.132  -0.920   0.022   0.552   0.426
E K  2NZ9A                165    0.290  59.653  -0.111   0.023   0.655   0.322
B S  2NZ9A                166    0.241  28.233  -1.722   0.021   0.756   0.223
B F  2NZ9A                167    0.100  20.170   0.640   0.023   0.655   0.322
E G  2NZ9A                168    0.304  23.956  -0.297   0.022   0.552   0.426
B H  2NZ9A                169    0.297  53.952  -0.507   0.022   0.552   0.426
E E  2NZ9A                170    0.449  78.370  -0.847   0.074   0.484   0.442
B V  2NZ9A                171    0.297  45.587  -1.430   0.069   0.386   0.545
E L  2NZ9A                172    0.375  68.717  -1.314   0.064   0.216   0.721
B N  2NZ9A                173    0.269  39.338  -0.940   0.113   0.087   0.800
B L  2NZ9A                174    0.203  37.243  -0.772   0.115   0.016   0.868
B T  2NZ9A                175    0.115  15.992  -1.493   0.257   0.016   0.727
E R  2NZ9A                176    0.334  76.394  -1.145   0.339   0.016   0.645
E N  2NZ9A                177    0.333  48.678  -0.229   0.257   0.016   0.727
B G  2NZ9A                178    0.088   6.910  -3.376   0.257   0.016   0.727
B Y  2NZ9A                179    0.117  25.067  -0.938   0.339   0.016   0.645
B G  2NZ9A                180    0.095   7.437  -2.752   0.455   0.046   0.498
B S  2NZ9A                181    0.065   7.595  -1.113   0.605   0.105   0.290
B T  2NZ9A                182    0.078  10.819  -0.391   0.725   0.163   0.112
B Q  2NZ9A                183    0.049   8.751   0.151   0.725   0.163   0.112
B Y  2NZ9A                184    0.094  20.088   0.094   0.662   0.225   0.113
B I  2NZ9A                185    0.030   5.494  -0.044   0.594   0.285   0.121
B R  2NZ9A                186    0.148  33.938   0.152   0.391   0.466   0.143
B F  2NZ9A                187    0.049   9.834  -0.519   0.252   0.423   0.325
B S  2NZ9A                188    0.078   9.165   0.249   0.125   0.227   0.648
B P  2NZ9A                189    0.157  22.278  -0.787   0.118   0.150   0.732
E D  2NZ9A                190    0.301  43.417  -0.850   0.125   0.227   0.648
B F  2NZ9A                191    0.094  18.846   0.444   0.148   0.418   0.435
B T  2NZ9A                192    0.181  25.035  -0.007   0.079   0.592   0.329
B F  2NZ9A                193    0.188  37.732  -0.053   0.087   0.683   0.230
E G  2NZ9A                194    0.284  22.374   0.170   0.087   0.683   0.230
B F  2NZ9A                195    0.094  18.906   0.099   0.079   0.592   0.329
E E  2NZ9A                196    0.444  77.619  -0.632   0.069   0.386   0.545
E E  2NZ9A                197    0.419  73.199  -1.234   0.125   0.227   0.648
B S  2NZ9A                198    0.306  35.840  -1.540   0.118   0.150   0.732
E L  2NZ9A                199    0.439  80.381  -1.260   0.118   0.150   0.732
E E  2NZ9A                200    0.594 103.772  -1.726   0.113   0.087   0.800
E V  2NZ9A                201    0.447  68.658  -1.704   0.113   0.087   0.800
E D  2NZ9A                202    0.467  67.237  -0.945   0.118   0.150   0.732
E T  2NZ9A                203    0.439  60.834  -1.745   0.118   0.150   0.732
E N  2NZ9A                204    0.394  57.623  -1.737   0.113   0.087   0.800
E P  2NZ9A                205    0.338  48.033  -1.726   0.199   0.152   0.649
E L  2NZ9A                206    0.321  58.775  -1.027   0.199   0.152   0.649
B L  2NZ9A                207    0.278  50.810  -1.403   0.191   0.086   0.723
E G  2NZ9A                208    0.377  29.678  -1.741   0.191   0.086   0.723
E A  2NZ9A                209    0.390  42.967  -0.750   0.278   0.093   0.628
E G  2NZ9A                210    0.358  28.159  -1.150   0.386   0.097   0.517
E K  2NZ9A                211    0.288  59.344   0.471   0.386   0.097   0.517
B F  2NZ9A                212    0.073  14.571  -0.088   0.502   0.102   0.396
B A  2NZ9A                213    0.057   6.325   0.361   0.428   0.171   0.402
B T  2NZ9A                214    0.113  15.673  -0.789   0.278   0.093   0.628
B D  2NZ9A                215    0.130  18.690   0.129   0.184   0.043   0.773
B P  2NZ9A                216    0.068   9.621   0.098   0.858   0.002   0.139
B A  2NZ9A                217    0.036   4.011  -0.865   0.970   0.001   0.030
B V  2NZ9A                218    0.040   6.117   0.122   0.970   0.001   0.030
B T  2NZ9A                219    0.097  13.440   0.815   0.988   0.000   0.012
B L  2NZ9A                220    0.025   4.523   0.814   0.988   0.000   0.012
B A  2NZ9A                221    0.024   2.667   0.808   0.988   0.000   0.012
B H  2NZ9A                222    0.127  23.174   0.466   0.970   0.001   0.030
B E  2NZ9A                223    0.059  10.360   0.152   0.988   0.000   0.012
B L  2NZ9A                224    0.021   3.863   1.084   0.970   0.001   0.030
B I  2NZ9A                225    0.028   5.217   0.493   0.970   0.001   0.030
B H  2NZ9A                226    0.175  31.923   0.696   0.970   0.001   0.030
B A  2NZ9A                227    0.041   4.540  -0.584   0.923   0.002   0.076
B G  2NZ9A                228    0.036   2.833  -0.208   0.879   0.010   0.111
B H  2NZ9A                229    0.130  23.720  -0.125   0.879   0.010   0.111
B R  2NZ9A                230    0.226  51.640  -0.909   0.717   0.014   0.269
B L  2NZ9A                231    0.048   8.862   0.118   0.561   0.047   0.393
B Y  2NZ9A                232    0.107  22.951   0.151   0.278   0.093   0.628
B G  2NZ9A                233    0.094   7.366  -1.985   0.125   0.227   0.648
B I  2NZ9A                234    0.063  11.729  -0.152   0.069   0.386   0.545
E A  2NZ9A                235    0.366  40.355  -0.002   0.021   0.451   0.528
B I  2NZ9A                236    0.223  41.255  -0.658   0.021   0.279   0.699
E N  2NZ9A                237    0.360  52.719  -0.432   0.019   0.141   0.840
E P  2NZ9A                238    0.515  73.135  -0.995   0.018   0.047   0.935
E N  2NZ9A                239    0.512  75.001  -0.936   0.018   0.047   0.935
E R  2NZ9A                240    0.577 132.202  -0.347   0.018   0.088   0.893
E V  2NZ9A                241    0.496  76.297  -1.393   0.019   0.141   0.840
B F  2NZ9A                242    0.153  30.767   0.333   0.020   0.205   0.775
E K  2NZ9A                243    0.434  89.212  -0.336   0.020   0.205   0.775
B V  2NZ9A                244    0.233  35.858   0.362   0.064   0.216   0.721
E N  2NZ9A                245    0.421  61.693  -0.585   0.064   0.216   0.721
E T  2NZ9A                246    0.356  49.322  -0.750   0.125   0.227   0.648
E N  2NZ9A                247    0.436  63.860  -0.888   0.125   0.227   0.648
B A  2NZ9A                248    0.216  23.858  -0.643   0.216   0.235   0.548
B Y  2NZ9A                249    0.278  59.430  -0.255   0.216   0.235   0.548
B Y  2NZ9A                250    0.237  50.668  -0.999   0.125   0.227   0.648
E E  2NZ9A                251    0.295  51.571  -1.160   0.125   0.227   0.648
B M  2NZ9A                252    0.256  51.166  -1.426   0.125   0.227   0.648
E S  2NZ9A                253    0.507  59.444  -1.274   0.118   0.150   0.732
E G  2NZ9A                254    0.556  43.757  -1.326   0.052   0.084   0.864
B L  2NZ9A                255    0.278  50.993   0.624   0.056   0.142   0.802
E E  2NZ9A                256    0.580 101.378   0.588   0.069   0.386   0.545
B V  2NZ9A                257    0.132  20.288   0.966   0.079   0.592   0.329
E S  2NZ9A                258    0.375  43.985   1.004   0.087   0.683   0.230
B F  2NZ9A                259    0.169  33.918   0.060   0.172   0.690   0.138
B E  2NZ9A                260    0.223  38.923   0.449   0.169   0.612   0.219
B E  2NZ9A                261    0.247  43.186   0.255   0.169   0.612   0.219
B L  2NZ9A                262    0.049   8.899   0.875   0.268   0.505   0.227
B R  2NZ9A                263    0.153  35.037   0.754   0.273   0.587   0.140
B T  2NZ9A                264    0.194  26.866  -0.890   0.159   0.516   0.325
B F  2NZ9A                265    0.150  30.025  -1.076   0.125   0.227   0.648
B G  2NZ9A                266    0.255  20.100  -1.617   0.018   0.088   0.893
B G  2NZ9A                267    0.231  18.219  -1.563   0.018   0.047   0.935
E H  2NZ9A                268    0.431  78.399  -0.708   0.018   0.088   0.893
E D  2NZ9A                269    0.330  47.611  -0.426   0.018   0.088   0.893
B A  2NZ9A                270    0.145  15.957   0.761   0.019   0.141   0.840
E K  2NZ9A                271    0.577 118.730   0.370   0.021   0.279   0.699
B F  2NZ9A                272    0.186  37.290   0.329   0.021   0.451   0.528
B I  2NZ9A                273    0.093  17.187   0.450   0.022   0.359   0.619
E D  2NZ9A                274    0.307  44.167   0.164   0.019   0.141   0.840
E S  2NZ9A                275    0.518  60.674  -0.386   0.184   0.043   0.773
E L  2NZ9A                276    0.533  97.501  -1.569   0.257   0.016   0.727
E Q  2NZ9A                277    0.327  58.474  -0.693   0.430   0.016   0.555
E E  2NZ9A                278    0.442  77.217  -0.824   0.502   0.002   0.495
E N  2NZ9A                279    0.404  59.190  -0.518   0.622   0.015   0.363
E E  2NZ9A                280    0.443  77.410  -0.052   0.717   0.014   0.269
B F  2NZ9A                281    0.074  14.852  -0.575   0.717   0.014   0.269
B R  2NZ9A                282    0.114  26.220   0.428   0.879   0.010   0.111
E L  2NZ9A                283    0.395  72.288  -0.045   0.970   0.001   0.030
E Y  2NZ9A                284    0.402  85.801   0.952   0.970   0.001   0.030
B Y  2NZ9A                285    0.039   8.270  -0.113   0.970   0.001   0.030
B Y  2NZ9A                286    0.062  13.185   0.047   0.970   0.001   0.030
E N  2NZ9A                287    0.592  86.625   0.561   0.970   0.001   0.030
E K  2NZ9A                288    0.333  68.478   1.041   0.923   0.002   0.076
B F  2NZ9A                289    0.040   8.088   0.382   0.923   0.002   0.076
E K  2NZ9A                290    0.391  80.367   1.616   0.970   0.001   0.030
E D  2NZ9A                291    0.526  75.753   1.331   0.970   0.001   0.030
B I  2NZ9A                292    0.042   7.733   0.575   0.970   0.001   0.030
B A  2NZ9A                293    0.058   6.425   0.178   0.970   0.001   0.030
E S  2NZ9A                294    0.572  67.015   1.297   0.970   0.001   0.030
E T  2NZ9A                295    0.272  37.754   1.331   0.923   0.002   0.076
B L  2NZ9A                296    0.063  11.517  -0.283   0.858   0.002   0.139
E N  2NZ9A                297    0.380  55.661  -0.377   0.802   0.014   0.185
E K  2NZ9A                298    0.552 113.464   0.519   0.622   0.015   0.363
B A  2NZ9A                299    0.158  17.401  -1.350   0.455   0.046   0.498
E K  2NZ9A                300    0.318  65.433  -0.989   0.278   0.093   0.628
E S  2NZ9A                301    0.482  56.490  -0.942   0.199   0.152   0.649
B I  2NZ9A                302    0.305  56.388  -1.662   0.199   0.152   0.649
E V  2NZ9A                303    0.374  57.561  -0.891   0.052   0.084   0.864
E G  2NZ9A                304    0.401  31.590  -2.010   0.018   0.019   0.964
E T  2NZ9A                305    0.553  76.687  -2.114   0.018   0.019   0.964
E T  2NZ9A                306    0.611  84.787  -1.921   0.018   0.019   0.964
B A  2NZ9A                307    0.170  18.778  -0.338   0.058   0.017   0.925
E S  2NZ9A                308    0.425  49.869   0.251   0.053   0.043   0.903
B L  2NZ9A                309    0.101  18.493  -0.182   0.522   0.016   0.462
E Q  2NZ9A                310    0.518  92.461   0.314   0.717   0.014   0.269
E Y  2NZ9A                311    0.384  81.975   1.327   0.858   0.002   0.139
B M  2NZ9A                312    0.065  12.906   0.037   0.923   0.002   0.076
B K  2NZ9A                313    0.198  40.667   0.515   0.923   0.002   0.076
E N  2NZ9A                314    0.573  83.902   0.526   0.975   0.003   0.022
B V  2NZ9A                315    0.230  35.412   1.362   0.938   0.007   0.055
B F  2NZ9A                316    0.031   6.202   0.324   0.938   0.007   0.055
E K  2NZ9A                317    0.384  79.071   1.389   0.938   0.007   0.055
E E  2NZ9A                318    0.580 101.256  -0.249   0.879   0.010   0.111
E K  2NZ9A                319    0.323  66.421   0.259   0.717   0.014   0.269
B Y  2NZ9A                320    0.178  38.081  -0.238   0.522   0.016   0.462
E L  2NZ9A                321    0.513  93.912  -0.147   0.184   0.043   0.773
B L  2NZ9A                322    0.196  35.888  -0.483   0.118   0.150   0.732
B S  2NZ9A                323    0.267  31.281  -1.081   0.064   0.216   0.721
E E  2NZ9A                324    0.561  98.077  -0.056   0.066   0.296   0.638
E D  2NZ9A                325    0.453  65.335  -1.041   0.056   0.142   0.802
B T  2NZ9A                326    0.295  40.986  -1.253   0.053   0.043   0.903
E S  2NZ9A                327    0.642  75.266  -1.005   0.053   0.043   0.903
E G  2NZ9A                328    0.363  28.560  -1.148   0.018   0.088   0.893
E K  2NZ9A                329    0.439  90.364   0.539   0.023   0.655   0.322
B F  2NZ9A                330    0.163  32.754   0.670   0.018   0.846   0.136
E S  2NZ9A                331    0.395  46.259   0.216   0.011   0.918   0.071
B V  2NZ9A                332    0.120  18.475   0.498   0.018   0.846   0.136
B D  2NZ9A                333    0.253  36.414   0.020   0.022   0.552   0.426
B K  2NZ9A                334    0.180  37.088   0.745   0.502   0.102   0.396
E L  2NZ9A                335    0.583 106.711  -0.796   0.622   0.015   0.363
E K  2NZ9A                336    0.274  56.465   0.967   0.717   0.014   0.269
B F  2NZ9A                337    0.038   7.627  -0.741   0.879   0.010   0.111
B D  2NZ9A                338    0.258  37.221   0.433   0.938   0.007   0.055
E K  2NZ9A                339    0.520 106.943   1.213   0.970   0.001   0.030
B L  2NZ9A                340    0.101  18.438   0.156   0.970   0.001   0.030
B Y  2NZ9A                341    0.058  12.480   0.726   0.970   0.001   0.030
E K  2NZ9A                342    0.460  94.725   0.893   0.923   0.002   0.076
E M  2NZ9A                343    0.396  79.220   0.514   0.923   0.002   0.076
B L  2NZ9A                344    0.077  14.062  -0.611   0.858   0.002   0.139
B T  2NZ9A                345    0.135  18.780   0.564   0.694   0.003   0.303
E E  2NZ9A                346    0.446  77.829  -1.411   0.502   0.002   0.495
E I  2NZ9A                347    0.334  61.864  -1.476   0.430   0.016   0.555
B Y  2NZ9A                348    0.198  42.206  -0.598   0.268   0.043   0.689
B T  2NZ9A                349    0.242  33.579  -0.832   0.268   0.043   0.689
E E  2NZ9A                350    0.373  65.233  -1.111   0.660   0.049   0.291
B D  2NZ9A                351    0.278  40.002  -0.492   0.751   0.050   0.199
E N  2NZ9A                352    0.380  55.559  -0.364   0.802   0.014   0.185
B F  2NZ9A                353    0.096  19.287  -0.313   0.879   0.010   0.111
B V  2NZ9A                354    0.209  32.185  -0.578   0.802   0.014   0.185
E K  2NZ9A                355    0.599 123.194   0.131   0.802   0.014   0.185
E F  2NZ9A                356    0.404  81.103   0.501   0.751   0.050   0.199
B F  2NZ9A                357    0.140  28.138   0.326   0.561   0.047   0.393
E K  2NZ9A                358    0.519 106.758   0.445   0.278   0.093   0.628
B V  2NZ9A                359    0.250  38.471  -0.092   0.307   0.165   0.527
E L  2NZ9A                360    0.487  89.243   0.024   0.307   0.165   0.527
B N  2NZ9A                361    0.238  34.814  -0.085   0.321   0.252   0.427
B R  2NZ9A                362    0.275  62.883   0.210   0.231   0.330   0.439
E K  2NZ9A                363    0.403  82.856  -0.163   0.252   0.423   0.325
B T  2NZ9A                364    0.295  40.875   0.681   0.268   0.505   0.227
B Y  2NZ9A                365    0.164  35.004   0.088   0.268   0.505   0.227
B L  2NZ9A                366    0.096  17.523   0.361   0.231   0.330   0.439
B N  2NZ9A                367    0.263  38.562  -0.587   0.216   0.235   0.548
E F  2NZ9A                368    0.393  78.915  -0.687   0.199   0.152   0.649
E D  2NZ9A                369    0.397  57.208  -1.309   0.118   0.150   0.732
B K  2NZ9A                370    0.232  47.702   0.176   0.113   0.087   0.800
B A  2NZ9A                371    0.242  26.635  -0.435   0.053   0.043   0.903
B V  2NZ9A                372    0.211  32.431  -1.420   0.118   0.150   0.732
E F  2NZ9A                373    0.332  66.652  -0.312   0.069   0.386   0.545
E K  2NZ9A                374    0.356  73.229  -0.036   0.074   0.484   0.442
B I  2NZ9A                375    0.131  24.216  -0.250   0.074   0.484   0.442
E N  2NZ9A                376    0.368  53.861  -1.055   0.069   0.386   0.545
B I  2NZ9A                377    0.143  26.455  -0.589   0.020   0.205   0.775
B V  2NZ9A                378    0.229  35.228  -0.544   0.019   0.141   0.840
E P  2NZ9A                379    0.517  73.348  -0.455   0.018   0.047   0.935
E K  2NZ9A                380    0.660 135.659  -1.576   0.018   0.019   0.964
E V  2NZ9A                381    0.544  83.582  -1.066   0.052   0.084   0.864
B N  2NZ9A                382    0.268  39.294  -0.165   0.069   0.386   0.545
B Y  2NZ9A                383    0.122  26.071   0.348   0.069   0.386   0.545
E T  2NZ9A                384    0.261  36.256   0.300   0.069   0.386   0.545
B I  2NZ9A                385    0.126  23.329   0.107   0.135   0.317   0.548
E Y  2NZ9A                386    0.348  74.346  -0.491   0.125   0.227   0.648
E D  2NZ9A                387    0.349  50.276  -0.705   0.113   0.087   0.800
B G  2NZ9A                388    0.131  10.325  -2.730   0.113   0.087   0.800
B F  2NZ9A                389    0.109  21.957  -0.319   0.125   0.227   0.648
B N  2NZ9A                390    0.202  29.602  -1.602   0.125   0.227   0.648
B L  2NZ9A                391    0.136  24.902  -0.243   0.118   0.150   0.732
E R  2NZ9A                392    0.367  83.951  -1.064   0.113   0.087   0.800
E N  2NZ9A                393    0.469  68.603  -0.858   0.191   0.086   0.723
E T  2NZ9A                394    0.431  59.807   0.327   0.199   0.152   0.649
E N  2NZ9A                395    0.383  56.144  -1.220   0.216   0.235   0.548
B L  2NZ9A                396    0.148  27.117   0.378   0.231   0.330   0.439
E A  2NZ9A                397    0.443  48.852   0.356   0.231   0.330   0.439
E A  2NZ9A                398    0.297  32.751  -0.078   0.231   0.330   0.439
E N  2NZ9A                399    0.415  60.697  -0.965   0.231   0.330   0.439
B F  2NZ9A                400    0.247  49.533  -1.135   0.125   0.227   0.648
E N  2NZ9A                401    0.486  71.121  -0.667   0.118   0.150   0.732
E G  2NZ9A                402    0.431  33.904  -2.396   0.113   0.087   0.800
E Q  2NZ9A                403    0.422  75.387  -0.159   0.118   0.150   0.732
B N  2NZ9A                404    0.308  45.033  -1.963   0.135   0.317   0.548
E T  2NZ9A                405    0.335  46.534   0.172   0.135   0.317   0.548
E E  2NZ9A                406    0.482  84.293  -1.536   0.135   0.317   0.548
E I  2NZ9A                407    0.262  48.415  -0.057   0.199   0.152   0.649
E N  2NZ9A                408    0.364  53.275  -0.913   0.191   0.086   0.723
E N  2NZ9A                409    0.435  63.743  -0.539   0.191   0.086   0.723
E M  2NZ9A                410    0.566 113.297  -0.163   0.191   0.086   0.723
B N  2NZ9A                411    0.185  27.099  -1.406   0.199   0.152   0.649
B F  2NZ9A                412    0.225  45.117  -1.567   0.125   0.227   0.648
E T  2NZ9A                413    0.521  72.304  -0.791   0.125   0.227   0.648
E K  2NZ9A                414    0.500 102.891   0.351   0.064   0.216   0.721
B L  2NZ9A                415    0.103  18.804  -0.537   0.056   0.142   0.802
E K  2NZ9A                416    0.571 117.434  -0.422   0.052   0.084   0.864
E N  2NZ9A                417    0.583  85.322  -1.538   0.113   0.043   0.844
E F  2NZ9A                418    0.351  70.546  -1.477   0.113   0.087   0.800
E T  2NZ9A                419    0.422  58.518  -1.029   0.118   0.150   0.732
B G  2NZ9A                420    0.203  15.952  -1.769   0.199   0.152   0.649
B L  2NZ9A                421    0.145  26.586  -0.720   0.252   0.423   0.325
B F  2NZ9A                422    0.140  28.018  -0.346   0.169   0.612   0.219
E E  2NZ9A                423    0.266  46.558  -0.017   0.172   0.690   0.138
B F  2NZ9A                424    0.149  29.884   0.038   0.172   0.690   0.138
B Y  2NZ9A                425    0.118  25.152  -0.606   0.169   0.612   0.219
B K  2NZ9A                426    0.198  40.729  -0.106   0.169   0.612   0.219
B L  2NZ9A                427    0.158  28.930  -0.800   0.169   0.612   0.219
B L  2NZ9A                428    0.126  23.144  -1.222   0.079   0.592   0.329
B C  2NZ9A                429    0.068   9.547  -2.101   0.079   0.592   0.329
B V  2NZ9A                430    0.192  29.526  -1.018   0.074   0.484   0.442
E R  2NZ9A                431    0.271  62.013  -0.289   0.022   0.359   0.619
B G  2NZ9A                432    0.281  22.138  -1.729   0.021   0.279   0.699
B I  2NZ9A                433    0.253  46.786  -1.300   0.021   0.451   0.528
B I  2NZ9A                434    0.196  36.186  -0.904   0.022   0.552   0.426
B T  2NZ9A                435    0.274  37.962  -0.793   0.021   0.279   0.699
E S  2NZ9A                436    0.505  59.174  -1.398   0.018   0.088   0.893
E K  2NZ9A                437    0.590 121.384  -0.990   0.018   0.019   0.964
E T  2NZ9A                438    0.461  63.899  -2.512   0.058   0.017   0.925
E K  2NZ9A                439    0.462  94.992  -1.466   0.113   0.043   0.844
E S  2NZ9A                440    0.313  36.707  -1.288   0.191   0.086   0.723
E L  2NZ9A                441    0.405  74.137  -0.818   0.278   0.093   0.628
E D  2NZ9A                442    0.369  53.144  -1.384   0.386   0.097   0.517
E K  2NZ9A                443    0.445  91.578  -1.182   0.386   0.097   0.517
E G  2NZ9A                444    0.330  26.002  -1.375   0.386   0.097   0.517
E Y  2NZ9A                445    0.347  74.261  -1.287   0.386   0.097   0.517
E N  2NZ9A                446    0.342  50.113  -1.426   0.386   0.097   0.517
E K  2NZ9A                447    0.480  98.798  -0.892   0.278   0.093   0.628
E A  2NZ9A                448    0.422  46.493  -1.615   0.278   0.093   0.628
B L  2NZ9A                449    0.282  51.707  -0.899   0.113   0.043   0.844
E N  2NZ9A                450    0.425  62.191  -1.310   0.053   0.043   0.903
B D  2NZ9A                451    0.283  40.751  -1.444   0.056   0.142   0.802
B L  2NZ9A                452    0.083  15.197  -0.306   0.021   0.451   0.528
B C  2NZ9A                453    0.040   5.560  -0.783   0.021   0.756   0.223
B I  2NZ9A                454    0.047   8.787   0.413   0.018   0.846   0.136
E K  2NZ9A                455    0.304  62.574   0.599   0.018   0.846   0.136
B V  2NZ9A                456    0.043   6.609   0.061   0.021   0.756   0.223
E N  2NZ9A                457    0.361  52.865   0.026   0.021   0.451   0.528
E N  2NZ9A                458    0.341  49.864  -1.029   0.018   0.088   0.893
E W  2NZ9A                459    0.416  99.952  -0.454   0.018   0.088   0.893
E D  2NZ9A                460    0.444  63.923  -0.888   0.020   0.205   0.775
B L  2NZ9A                461    0.097  17.816   0.413   0.022   0.359   0.619
B F  2NZ9A                462    0.124  24.827  -0.087   0.023   0.655   0.322
B F  2NZ9A                463    0.112  22.498  -0.645   0.021   0.756   0.223
B S  2NZ9A                464    0.099  11.615  -0.858   0.021   0.756   0.223
B P  2NZ9A                465    0.153  21.753  -1.246   0.021   0.451   0.528
E S  2NZ9A                466    0.437  51.240  -0.891   0.019   0.141   0.840
E E  2NZ9A                467    0.502  87.752  -0.949   0.053   0.043   0.903
E D  2NZ9A                468    0.524  75.465  -2.149   0.053   0.043   0.903
E N  2NZ9A                469    0.328  48.048  -1.735   0.113   0.043   0.844
B F  2NZ9A                470    0.283  56.798  -0.776   0.184   0.043   0.773
E T  2NZ9A                471    0.442  61.264  -0.938   0.184   0.043   0.773
E N  2NZ9A                472    0.631  92.305  -0.203   0.184   0.043   0.773
E D  2NZ9A                473    0.442  63.620  -1.025   0.184   0.043   0.773
B L  2NZ9A                474    0.147  26.842  -0.259   0.184   0.043   0.773
E N  2NZ9A                475    0.296  43.305  -0.822   0.184   0.043   0.773
E K  2NZ9A                476    0.550 113.135   0.668   0.113   0.043   0.844
E G  2NZ9A                477    0.481  37.831  -0.665   0.052   0.084   0.864
E E  2NZ9A                478    0.402  70.299   0.572   0.066   0.296   0.638
E E  2NZ9A                479    0.571  99.666   1.008   0.021   0.451   0.528
B I  2NZ9A                480    0.082  15.096   0.483   0.021   0.279   0.699
E T  2NZ9A                481    0.549  76.119  -0.389   0.064   0.216   0.721
E S  2NZ9A                482    0.405  47.501  -0.534   0.118   0.150   0.732
E D  2NZ9A                483    0.516  74.356  -1.170   0.118   0.150   0.732
E T  2NZ9A                484    0.363  50.376  -0.241   0.118   0.150   0.732
E N  2NZ9A                485    0.483  70.740  -0.540   0.125   0.227   0.648
E I  2NZ9A                486    0.344  63.603  -0.138   0.135   0.317   0.548
E E  2NZ9A                487    0.423  73.933  -0.475   0.069   0.386   0.545
E A  2NZ9A                488    0.434  47.783  -0.648   0.069   0.386   0.545
B A  2NZ9A                489    0.176  19.439   0.182   0.064   0.216   0.721
E E  2NZ9A                490    0.559  97.657  -1.140   0.056   0.142   0.802
E E  2NZ9A                491    0.420  73.391  -1.677   0.113   0.087   0.800
B N  2NZ9A                492    0.275  40.260  -1.653   0.191   0.086   0.723
B I  2NZ9A                493    0.258  47.804  -0.910   0.191   0.086   0.723
B S  2NZ9A                494    0.294  34.457  -1.414   0.278   0.093   0.628
B L  2NZ9A                495    0.267  48.851  -0.686   0.278   0.093   0.628
E D  2NZ9A                496    0.359  51.660  -1.383   0.268   0.043   0.689
B L  2NZ9A                497    0.280  51.341  -0.393   0.268   0.043   0.689
B I  2NZ9A                498    0.186  34.354  -1.028   0.386   0.097   0.517
B Q  2NZ9A                499    0.257  45.918  -0.755   0.386   0.097   0.517
E Q  2NZ9A                500    0.393  70.226  -0.696   0.386   0.097   0.517
B Y  2NZ9A                501    0.173  36.927  -0.262   0.538   0.173   0.289
B Y  2NZ9A                502    0.097  20.729   0.319   0.428   0.171   0.402
B L  2NZ9A                503    0.142  25.964  -0.291   0.321   0.252   0.427
B T  2NZ9A                504    0.273  37.810  -0.852   0.216   0.235   0.548
B F  2NZ9A                505    0.325  65.187  -0.875   0.125   0.227   0.648
E N  2NZ9A                506    0.525  76.816  -0.435   0.056   0.142   0.802
B F  2NZ9A                507    0.151  30.266   0.131   0.056   0.142   0.802
E D  2NZ9A                508    0.624  89.933  -1.119   0.056   0.142   0.802
E N  2NZ9A                509    0.381  55.720  -1.135   0.052   0.084   0.864
B E  2NZ9A                510    0.242  42.347  -0.397   0.053   0.043   0.903
B P  2NZ9A                511    0.258  36.624  -1.538   0.053   0.043   0.903
E E  2NZ9A                512    0.522  91.263  -0.862   0.053   0.043   0.903
E N  2NZ9A                513    0.444  64.972  -1.528   0.052   0.084   0.864
B I  2NZ9A                514    0.212  39.294  -1.068   0.056   0.142   0.802
E S  2NZ9A                515    0.499  58.506  -1.086   0.056   0.142   0.802
E I  2NZ9A                516    0.373  68.912  -1.472   0.052   0.084   0.864
E E  2NZ9A                517    0.573 100.156  -0.631   0.052   0.084   0.864
E N  2NZ9A                518    0.494  72.263  -0.397   0.052   0.084   0.864
B L  2NZ9A                519    0.120  21.899  -0.406   0.053   0.043   0.903
E S  2NZ9A                520    0.293  34.340  -0.146   0.053   0.043   0.903
E S  2NZ9A                521    0.346  40.504  -0.997   0.113   0.087   0.800
E D  2NZ9A                522    0.382  55.003  -1.038   0.113   0.087   0.800
B I  2NZ9A                523    0.260  48.137  -0.288   0.113   0.087   0.800
E I  2NZ9A                524    0.582 107.614  -0.989   0.113   0.087   0.800
E G  2NZ9A                525    0.424  33.345  -1.361   0.113   0.087   0.800
E Q  2NZ9A                526    0.480  85.639  -1.470   0.052   0.084   0.864
E L  2NZ9A                527    0.403  73.863  -1.821   0.056   0.142   0.802
E E  2NZ9A                528    0.396  69.129  -0.664   0.021   0.279   0.699
B L  2NZ9A                529    0.240  43.871  -0.632   0.021   0.451   0.528
B M  2NZ9A                530    0.114  22.731   0.845   0.022   0.359   0.619
B P  2NZ9A                531    0.243  34.538  -1.032   0.021   0.279   0.699
E N  2NZ9A                532    0.297  43.510  -0.620   0.021   0.279   0.699
E I  2NZ9A                533    0.345  63.751  -0.613   0.022   0.359   0.619
E E  2NZ9A                534    0.535  93.395  -0.603   0.022   0.359   0.619
E R  2NZ9A                535    0.536 122.744  -0.295   0.021   0.279   0.699
B F  2NZ9A                536    0.213  42.809  -0.045   0.066   0.296   0.638
E P  2NZ9A                537    0.494  70.070  -0.850   0.066   0.296   0.638
E N  2NZ9A                538    0.653  95.658  -0.177   0.056   0.142   0.802
B G  2NZ9A                539    0.268  21.068  -0.556   0.020   0.205   0.775
E K  2NZ9A                540    0.479  98.592   0.498   0.021   0.451   0.528
E K  2NZ9A                541    0.640 131.730   0.890   0.021   0.756   0.223
B Y  2NZ9A                542    0.183  39.022   1.579   0.021   0.756   0.223
E E  2NZ9A                543    0.429  75.016  -0.780   0.023   0.655   0.322
B L  2NZ9A                544    0.122  22.338  -0.456   0.021   0.451   0.528
E D  2NZ9A                545    0.410  59.038  -0.831   0.019   0.141   0.840
E K  2NZ9A                546    0.429  88.307  -0.141   0.052   0.084   0.864
B Y  2NZ9A                547    0.233  49.707  -0.870   0.113   0.043   0.844
B T  2NZ9A                548    0.231  31.998  -0.209   0.184   0.043   0.773
B M  2NZ9A                549    0.079  15.828  -1.254   0.428   0.171   0.402
B F  2NZ9A                550    0.118  23.622  -0.140   0.453   0.248   0.299
B H  2NZ9A                551    0.160  29.122  -0.060   0.352   0.332   0.316
B Y  2NZ9A                552    0.082  17.566   0.602   0.373   0.401   0.226
B L  2NZ9A                553    0.059  10.803   0.316   0.373   0.401   0.226
B R  2NZ9A                554    0.203  46.441   0.372   0.252   0.423   0.325
B A  2NZ9A                555    0.047   5.168  -1.826   0.252   0.423   0.325
B Q  2NZ9A                556    0.201  35.934   0.009   0.159   0.516   0.325
E E  2NZ9A                557    0.487  85.114   0.219   0.148   0.418   0.435
B F  2NZ9A                558    0.170  34.179   0.640   0.056   0.142   0.802
E E  2NZ9A                559    0.494  86.354  -0.865   0.052   0.084   0.864
E H  2NZ9A                560    0.575 104.647  -0.761   0.053   0.043   0.903
E G  2NZ9A                561    0.514  40.491  -1.302   0.053   0.043   0.903
E K  2NZ9A                562    0.413  85.036  -1.089   0.052   0.084   0.864
E S  2NZ9A                563    0.571  66.874  -0.498   0.056   0.142   0.802
E R  2NZ9A                564    0.368  84.341   0.307   0.069   0.386   0.545
B I  2NZ9A                565    0.068  12.488   0.402   0.023   0.655   0.322
E A  2NZ9A                566    0.340  37.490   0.499   0.021   0.756   0.223
B L  2NZ9A                567    0.087  16.021   0.270   0.087   0.683   0.230
B T  2NZ9A                568    0.154  21.401  -0.512   0.074   0.484   0.442
E N  2NZ9A                569    0.340  49.776  -0.887   0.216   0.235   0.548
E S  2NZ9A                570    0.298  34.867  -0.039   0.455   0.046   0.498
B V  2NZ9A                571    0.051   7.839   0.222   0.879   0.010   0.111
B N  2NZ9A                572    0.238  34.785   0.147   0.879   0.010   0.111
E E  2NZ9A                573    0.442  77.130   1.109   0.879   0.010   0.111
B A  2NZ9A                574    0.080   8.794  -0.756   0.879   0.010   0.111
B L  2NZ9A                575    0.066  12.011   0.062   0.802   0.014   0.185
E L  2NZ9A                576    0.429  78.458   0.186   0.522   0.016   0.462
E N  2NZ9A                577    0.477  69.891  -0.946   0.115   0.016   0.868
E P  2NZ9A                578    0.599  84.984   0.124   0.018   0.019   0.964
E S  2NZ9A                579    0.311  36.426  -1.194   0.018   0.047   0.935
B R  2NZ9A                580    0.216  49.578   0.934   0.021   0.451   0.528
B V  2NZ9A                581    0.070  10.820  -0.155   0.018   0.846   0.136
B Y  2NZ9A                582    0.098  20.878   0.720   0.018   0.846   0.136
B T  2NZ9A                583    0.097  13.440  -1.037   0.018   0.846   0.136
B F  2NZ9A                584    0.087  17.561  -0.258   0.023   0.655   0.322
B F  2NZ9A                585    0.097  19.388   0.225   0.022   0.359   0.619
B S  2NZ9A                586    0.236  27.647  -0.403   0.018   0.088   0.893
B S  2NZ9A                587    0.302  35.430  -1.419   0.184   0.043   0.773
E D  2NZ9A                588    0.514  74.111  -0.860   0.268   0.043   0.689
B Y  2NZ9A                589    0.187  39.962  -0.255   0.386   0.097   0.517
B V  2NZ9A                590    0.068  10.390  -0.447   0.502   0.102   0.396
E K  2NZ9A                591    0.559 115.007   0.652   0.455   0.046   0.498
E K  2NZ9A                592    0.528 108.671   0.561   0.354   0.048   0.598
B V  2NZ9A                593    0.254  39.086  -1.034   0.181   0.016   0.803
E N  2NZ9A                594    0.405  59.292  -0.856   0.058   0.017   0.925
E K  2NZ9A                595    0.472  97.152   0.900   0.181   0.016   0.803
B A  2NZ9A                596    0.165  18.194  -1.226   0.430   0.016   0.555
B T  2NZ9A                597    0.144  20.001  -0.890   0.430   0.016   0.555
B E  2NZ9A                598    0.224  39.063  -0.382   0.622   0.015   0.363
B A  2NZ9A                599    0.061   6.678  -1.569   0.879   0.010   0.111
B A  2NZ9A                600    0.207  22.822  -0.145   0.938   0.007   0.055
B M  2NZ9A                601    0.153  30.515   0.842   0.970   0.001   0.030
B F  2NZ9A                602    0.033   6.663   0.388   0.970   0.001   0.030
B L  2NZ9A                603    0.070  12.890   0.269   0.988   0.000   0.012
E G  2NZ9A                604    0.252  19.825   0.002   0.988   0.000   0.012
B W  2NZ9A                605    0.058  13.997   0.331   0.988   0.000   0.012
B V  2NZ9A                606    0.023   3.581   0.545   0.988   0.000   0.012
E E  2NZ9A                607    0.297  51.816   0.977   0.970   0.001   0.030
E Q  2NZ9A                608    0.366  65.439   0.913   0.970   0.001   0.030
B L  2NZ9A                609    0.034   6.207   0.526   0.970   0.001   0.030
B V  2NZ9A                610    0.038   5.917  -0.016   0.938   0.007   0.055
E Y  2NZ9A                611    0.426  91.058   0.988   0.879   0.010   0.111
E D  2NZ9A                612    0.315  45.392  -0.472   0.717   0.014   0.269
B F  2NZ9A                613    0.080  16.076   0.897   0.522   0.016   0.462
B T  2NZ9A                614    0.196  27.144  -0.356   0.354   0.048   0.598
E D  2NZ9A                615    0.364  52.452  -0.504   0.354   0.048   0.598
E E  2NZ9A                616    0.486  84.852  -0.278   0.268   0.043   0.689
B T  2NZ9A                617    0.155  21.429  -0.606   0.268   0.043   0.689
E S  2NZ9A                618    0.381  44.665  -0.746   0.278   0.093   0.628
E E  2NZ9A                619    0.478  83.559   0.177   0.307   0.165   0.527
E V  2NZ9A                620    0.496  76.174   0.757   0.216   0.235   0.548
E S  2NZ9A                621    0.413  48.357   0.262   0.216   0.235   0.548
E T  2NZ9A                622    0.499  69.156  -0.085   0.231   0.330   0.439
B T  2NZ9A                623    0.176  24.397   0.244   0.216   0.235   0.548
E D  2NZ9A                624    0.379  54.571  -0.153   0.199   0.152   0.649
E K  2NZ9A                625    0.532 109.474   0.655   0.307   0.165   0.527
E I  2NZ9A                626    0.337  62.345  -1.306   0.307   0.165   0.527
B A  2NZ9A                627    0.147  16.221   0.351   0.113   0.087   0.800
E D  2NZ9A                628    0.455  65.638  -0.042   0.113   0.087   0.800
B I  2NZ9A                629    0.083  15.373   0.318   0.066   0.296   0.638
E T  2NZ9A                630    0.308  42.789  -0.643   0.079   0.592   0.329
B I  2NZ9A                631    0.181  33.393   0.590   0.085   0.775   0.140
B I  2NZ9A                632    0.076  14.060  -0.157   0.085   0.775   0.140
B I  2NZ9A                633    0.049   9.139   1.258   0.079   0.592   0.329
B P  2NZ9A                634    0.161  22.789  -0.410   0.148   0.418   0.435
B Y  2NZ9A                635    0.139  29.768   0.015   0.135   0.317   0.548
B I  2NZ9A                636    0.094  17.353  -0.571   0.231   0.330   0.439
B G  2NZ9A                637    0.138  10.861  -0.322   0.199   0.152   0.649
E P  2NZ9A                638    0.283  40.172  -0.076   0.278   0.093   0.628
B A  2NZ9A                639    0.055   6.072  -1.044   0.386   0.097   0.517
B L  2NZ9A                640    0.090  16.461  -0.052   0.428   0.171   0.402
B N  2NZ9A                641    0.182  26.586  -1.129   0.307   0.165   0.527
B I  2NZ9A                642    0.059  10.970  -0.391   0.307   0.165   0.527
B G  2NZ9A                643    0.106   8.311  -1.084   0.307   0.165   0.527
B N  2NZ9A                644    0.267  39.103  -0.522   0.307   0.165   0.527
E M  2NZ9A                645    0.322  64.372  -0.697   0.278   0.093   0.628
B L  2NZ9A                646    0.278  50.883  -0.969   0.191   0.086   0.723
E Y  2NZ9A                647    0.393  84.027  -0.472   0.191   0.086   0.723
E K  2NZ9A                648    0.543 111.716  -0.237   0.184   0.043   0.773
E D  2NZ9A                649    0.525  75.580  -1.908   0.181   0.016   0.803
E D  2NZ9A                650    0.351  50.651  -0.244   0.257   0.016   0.727
B F  2NZ9A                651    0.066  13.186   0.333   0.660   0.049   0.291
E V  2NZ9A                652    0.329  50.598   1.421   0.911   0.033   0.057
E G  2NZ9A                653    0.418  32.928   0.218   0.938   0.007   0.055
B A  2NZ9A                654    0.095  10.436   0.257   0.938   0.007   0.055
B L  2NZ9A                655    0.051   9.247   0.720   0.879   0.010   0.111
E I  2NZ9A                656    0.422  78.070   0.696   0.879   0.010   0.111
E F  2NZ9A                657    0.312  62.638  -0.372   0.717   0.014   0.269
B S  2NZ9A                658    0.105  12.259  -0.083   0.430   0.016   0.555
B G  2NZ9A                659    0.117   9.216  -1.192   0.181   0.016   0.803
B A  2NZ9A                660    0.072   7.912  -0.451   0.502   0.102   0.396
B V  2NZ9A                661    0.104  16.031  -1.178   0.662   0.225   0.113
B I  2NZ9A                662    0.091  16.743   0.265   0.578   0.229   0.194
B L  2NZ9A                663    0.047   8.697   0.165   0.578   0.229   0.194
B L  2NZ9A                664    0.061  11.242   0.478   0.538   0.173   0.289
E E  2NZ9A                665    0.370  64.674  -0.101   0.428   0.171   0.402
E F  2NZ9A                666    0.282  56.658  -0.346   0.278   0.093   0.628
B I  2NZ9A                667    0.241  44.585   0.679   0.113   0.043   0.844
E P  2NZ9A                668    0.389  55.157  -0.965   0.113   0.043   0.844
E E  2NZ9A                669    0.419  73.164  -0.676   0.184   0.043   0.773
B I  2NZ9A                670    0.087  16.040   0.306   0.191   0.086   0.723
B A  2NZ9A                671    0.169  18.591  -0.190   0.278   0.093   0.628
B I  2NZ9A                672    0.103  19.055   0.859   0.386   0.097   0.517
B P  2NZ9A                673    0.176  24.946   0.075   0.455   0.046   0.498
B V  2NZ9A                674    0.166  25.545   0.149   0.605   0.105   0.290
B L  2NZ9A                675    0.072  13.128   0.196   0.649   0.163   0.188
B G  2NZ9A                676    0.140  11.010  -0.357   0.538   0.173   0.289
B T  2NZ9A                677    0.143  19.862  -0.305   0.578   0.229   0.194
B F  2NZ9A                678    0.147  29.563   0.314   0.453   0.248   0.299
B A  2NZ9A                679    0.180  19.803   0.175   0.352   0.332   0.316
B L  2NZ9A                680    0.145  26.476  -0.028   0.352   0.332   0.316
B V  2NZ9A                681    0.249  38.333   0.160   0.252   0.423   0.325
E S  2NZ9A                682    0.383  44.899  -1.197   0.148   0.418   0.435
E Y  2NZ9A                683    0.349  74.603  -0.213   0.125   0.227   0.648
B I  2NZ9A                684    0.082  15.207  -0.101   0.118   0.150   0.732
E A  2NZ9A                685    0.490  54.020  -0.229   0.191   0.086   0.723
E N  2NZ9A                686    0.560  82.043  -1.662   0.184   0.043   0.773
E K  2NZ9A                687    0.456  93.779  -0.059   0.354   0.048   0.598
E V  2NZ9A                688    0.385  59.144  -1.531   0.354   0.048   0.598
E L  2NZ9A                689    0.333  60.936   0.564   0.561   0.047   0.393
B T  2NZ9A                690    0.109  15.077  -0.421   0.701   0.107   0.192
B V  2NZ9A                691    0.067  10.236   0.582   0.701   0.107   0.192
E Q  2NZ9A                692    0.372  66.421   0.687   0.779   0.100   0.120
B T  2NZ9A                693    0.132  18.322   0.022   0.831   0.044   0.125
B I  2NZ9A                694    0.087  16.132  -0.002   0.879   0.010   0.111
B D  2NZ9A                695    0.275  39.570  -0.444   0.879   0.010   0.111
E N  2NZ9A                696    0.368  53.817  -0.105   0.923   0.002   0.076
B A  2NZ9A                697    0.057   6.226  -0.003   0.923   0.002   0.076
B L  2NZ9A                698    0.096  17.523   0.828   0.923   0.002   0.076
E S  2NZ9A                699    0.487  57.088   0.938   0.923   0.002   0.076
E K  2NZ9A                700    0.499 102.747   0.919   0.858   0.002   0.139
B R  2NZ9A                701    0.145  33.159   0.023   0.858   0.002   0.139
B N  2NZ9A                702    0.185  27.157   0.177   0.923   0.002   0.076
E E  2NZ9A                703    0.585 102.130   0.984   0.923   0.002   0.076
E K  2NZ9A                704    0.264  54.325   1.350   0.923   0.002   0.076
B W  2NZ9A                705    0.020   4.906   1.132   0.970   0.001   0.030
E D  2NZ9A                706    0.306  44.066   1.229   0.970   0.001   0.030
E E  2NZ9A                707    0.484  84.572   0.735   0.970   0.001   0.030
B V  2NZ9A                708    0.040   6.179   0.026   0.970   0.001   0.030
B Y  2NZ9A                709    0.053  11.347   0.706   0.970   0.001   0.030
E K  2NZ9A                710    0.323  66.379   0.209   0.970   0.001   0.030
B Y  2NZ9A                711    0.218  46.608   0.377   0.970   0.001   0.030
B I  2NZ9A                712    0.041   7.622   0.481   0.970   0.001   0.030
B V  2NZ9A                713    0.066  10.113  -0.172   0.970   0.001   0.030
B T  2NZ9A                714    0.178  24.702  -1.348   0.923   0.002   0.076
B N  2NZ9A                715    0.184  26.894  -0.072   0.923   0.002   0.076
B W  2NZ9A                716    0.061  14.670  -0.266   0.970   0.001   0.030
B L  2NZ9A                717    0.066  12.158  -0.050   0.970   0.001   0.030
B A  2NZ9A                718    0.127  14.006  -0.621   0.970   0.001   0.030
E K  2NZ9A                719    0.233  48.010   0.781   0.970   0.001   0.030
B V  2NZ9A                720    0.041   6.256   0.185   0.970   0.001   0.030
B N  2NZ9A                721    0.108  15.797   0.478   0.923   0.002   0.076
B T  2NZ9A                722    0.161  22.386  -0.345   0.923   0.002   0.076
B Q  2NZ9A                723    0.120  21.396   0.065   0.923   0.002   0.076
B I  2NZ9A                724    0.045   8.325  -0.009   0.970   0.001   0.030
B D  2NZ9A                725    0.203  29.238  -0.145   0.970   0.001   0.030
E L  2NZ9A                726    0.379  69.395   0.556   0.970   0.001   0.030
B I  2NZ9A                727    0.107  19.739   0.188   0.988   0.000   0.012
B R  2NZ9A                728    0.103  23.518   0.517   0.988   0.000   0.012
B K  2NZ9A                729    0.223  45.809   0.495   0.988   0.000   0.012
B K  2NZ9A                730    0.187  38.445   0.590   0.970   0.001   0.030
B M  2NZ9A                731    0.028   5.523   0.025   0.970   0.001   0.030
B K  2NZ9A                732    0.180  37.026   0.037   0.970   0.001   0.030
E E  2NZ9A                733    0.403  70.387   0.852   0.970   0.001   0.030
B A  2NZ9A                734    0.055   6.050  -0.795   0.970   0.001   0.030
B L  2NZ9A                735    0.045   8.240  -0.110   0.970   0.001   0.030
E E  2NZ9A                736    0.265  46.313   0.904   0.970   0.001   0.030
B N  2NZ9A                737    0.251  36.732   0.119   0.923   0.002   0.076
B Q  2NZ9A                738    0.080  14.234  -0.206   0.970   0.001   0.030
B A  2NZ9A                739    0.111  12.276  -0.877   0.970   0.001   0.030
E E  2NZ9A                740    0.549  95.980   1.232   0.970   0.001   0.030
B A  2NZ9A                741    0.069   7.549   0.279   0.988   0.000   0.012
B T  2NZ9A                742    0.024   3.343   0.929   0.988   0.000   0.012
E K  2NZ9A                743    0.328  67.531   1.793   0.988   0.000   0.012
E A  2NZ9A                744    0.405  44.653   1.978   0.988   0.000   0.012
B I  2NZ9A                745    0.071  13.154   0.956   0.988   0.000   0.012
B I  2NZ9A                746    0.029   5.384   0.679   0.970   0.001   0.030
E N  2NZ9A                747    0.434  63.582   1.495   0.970   0.001   0.030
E Y  2NZ9A                748    0.391  83.642   0.657   0.923   0.002   0.076
B Q  2NZ9A                749    0.246  43.936   0.706   0.923   0.002   0.076
B Y  2NZ9A                750    0.095  20.216   0.608   0.858   0.002   0.139
E N  2NZ9A                751    0.516  75.484   0.544   0.782   0.003   0.216
E Q  2NZ9A                752    0.475  84.817   0.934   0.717   0.014   0.269
B Y  2NZ9A                753    0.159  33.978   0.170   0.430   0.016   0.555
E T  2NZ9A                754    0.346  47.990  -0.837   0.339   0.016   0.645
E E  2NZ9A                755    0.548  95.753  -0.615   0.430   0.016   0.555
E E  2NZ9A                756    0.656 114.673  -0.425   0.339   0.016   0.645
E E  2NZ9A                757    0.425  74.230  -0.513   0.430   0.016   0.555
E K  2NZ9A                758    0.404  83.144  -0.479   0.522   0.016   0.462
E N  2NZ9A                759    0.558  81.691  -1.079   0.522   0.016   0.462
E N  2NZ9A                760    0.460  67.329  -0.351   0.430   0.016   0.555
B I  2NZ9A                761    0.114  21.016  -0.554   0.522   0.016   0.462
E N  2NZ9A                762    0.508  74.371   0.328   0.858   0.002   0.139
E F  2NZ9A                763    0.410  82.227  -0.114   0.923   0.002   0.076
B N  2NZ9A                764    0.223  32.647   0.070   0.923   0.002   0.076
B I  2NZ9A                765    0.070  12.932  -0.451   0.970   0.001   0.030
E D  2NZ9A                766    0.426  61.343   0.486   0.923   0.002   0.076
E D  2NZ9A                767    0.472  68.015   0.830   0.923   0.002   0.076
B L  2NZ9A                768    0.050   9.082   0.233   0.858   0.002   0.139
E S  2NZ9A                769    0.360  42.215   0.694   0.858   0.002   0.139
E S  2NZ9A                770    0.542  63.499  -0.259   0.858   0.002   0.139
E K  2NZ9A                771    0.495 101.801   1.238   0.858   0.002   0.139
B L  2NZ9A                772    0.076  13.952  -0.458   0.923   0.002   0.076
E N  2NZ9A                773    0.380  55.676  -0.005   0.858   0.002   0.139
E E  2NZ9A                774    0.582 101.763   0.172   0.923   0.002   0.076
E S  2NZ9A                775    0.338  39.567   0.487   0.970   0.001   0.030
B I  2NZ9A                776    0.040   7.326  -0.784   0.970   0.001   0.030
E N  2NZ9A                777    0.335  49.000   0.181   0.970   0.001   0.030
E K  2NZ9A                778    0.329  67.716   1.243   0.970   0.001   0.030
B A  2NZ9A                779    0.039   4.254  -1.149   0.970   0.001   0.030
B M  2NZ9A                780    0.070  13.987  -0.436   0.923   0.002   0.076
E I  2NZ9A                781    0.367  67.895   0.203   0.923   0.002   0.076
B N  2NZ9A                782    0.184  26.938   0.055   0.858   0.002   0.139
B I  2NZ9A                783    0.026   4.884   0.440   0.923   0.002   0.076
B N  2NZ9A                784    0.201  29.397  -0.280   0.858   0.002   0.139
E K  2NZ9A                785    0.378  77.796   1.081   0.923   0.002   0.076
B F  2NZ9A                786    0.052  10.497   0.108   0.923   0.002   0.076
B L  2NZ9A                787    0.051   9.301   0.407   0.858   0.002   0.139
E N  2NZ9A                788    0.256  37.434   0.218   0.858   0.002   0.139
E Q  2NZ9A                789    0.318  56.866   0.378   0.858   0.002   0.139
B C  2NZ9A                790    0.048   6.753  -0.269   0.802   0.014   0.185
B S  2NZ9A                791    0.054   6.305  -0.395   0.879   0.010   0.111
B V  2NZ9A                792    0.075  11.497   0.070   0.938   0.007   0.055
B S  2NZ9A                793    0.108  12.669   0.115   0.938   0.007   0.055
B Y  2NZ9A                794    0.120  25.665   0.917   0.938   0.007   0.055
B L  2NZ9A                795    0.065  11.810  -0.793   0.938   0.007   0.055
B M  2NZ9A                796    0.139  27.814   0.580   0.879   0.010   0.111
E N  2NZ9A                797    0.490  71.736   0.114   0.802   0.014   0.185
E S  2NZ9A                798    0.382  44.759  -0.258   0.600   0.003   0.397
B M  2NZ9A                799    0.087  17.489  -0.384   0.502   0.002   0.495
B I  2NZ9A                800    0.143  26.399   1.031   0.406   0.004   0.590
E P  2NZ9A                801    0.457  64.877  -0.076   0.923   0.002   0.076
E Y  2NZ9A                802    0.432  92.340   0.912   0.970   0.001   0.030
B G  2NZ9A                803    0.087   6.815  -0.100   0.970   0.001   0.030
B V  2NZ9A                804    0.072  11.005   0.528   0.970   0.001   0.030
E K  2NZ9A                805    0.577 118.709   0.945   0.970   0.001   0.030
E R  2NZ9A                806    0.261  59.769   1.624   0.970   0.001   0.030
B L  2NZ9A                807    0.025   4.614   0.547   0.970   0.001   0.030
E E  2NZ9A                808    0.400  69.810   1.295   0.970   0.001   0.030
E D  2NZ9A                809    0.546  78.736   1.764   0.970   0.001   0.030
B F  2NZ9A                810    0.046   9.172   0.526   0.923   0.002   0.076
B D  2NZ9A                811    0.091  13.113  -0.085   0.858   0.002   0.139
E A  2NZ9A                812    0.585  64.500   1.109   0.923   0.002   0.076
E S  2NZ9A                813    0.428  50.150   1.210   0.923   0.002   0.076
B L  2NZ9A                814    0.033   6.006   0.490   0.923   0.002   0.076
B K  2NZ9A                815    0.196  40.214   0.638   0.970   0.001   0.030
E D  2NZ9A                816    0.539  77.684   1.450   0.970   0.001   0.030
B A  2NZ9A                817    0.212  23.318   1.005   0.970   0.001   0.030
B L  2NZ9A                818    0.029   5.347   0.572   0.970   0.001   0.030
B L  2NZ9A                819    0.152  27.776   0.750   0.970   0.001   0.030
E K  2NZ9A                820    0.506 104.002   1.189   0.923   0.002   0.076
B Y  2NZ9A                821    0.061  13.078   0.952   0.923   0.002   0.076
B I  2NZ9A                822    0.026   4.791   0.263   0.938   0.007   0.055
E Y  2NZ9A                823    0.364  77.701   0.465   0.879   0.010   0.111
E D  2NZ9A                824    0.509  73.405   0.203   0.694   0.003   0.303
E N  2NZ9A                825    0.295  43.247  -0.241   0.321   0.003   0.675
E R  2NZ9A                826    0.381  87.203   0.137   0.115   0.016   0.868
B G  2NZ9A                827    0.160  12.592  -0.183   0.056   0.142   0.802
B T  2NZ9A                828    0.188  26.020  -0.125   0.069   0.386   0.545
B L  2NZ9A                829    0.146  26.733  -0.310   0.069   0.386   0.545
B I  2NZ9A                830    0.149  27.528  -0.351   0.125   0.227   0.648
E G  2NZ9A                831    0.494  38.870  -1.010   0.113   0.087   0.800
E Q  2NZ9A                832    0.363  64.796  -0.298   0.455   0.046   0.498
B V  2NZ9A                833    0.251  38.548  -0.087   0.802   0.014   0.185
E D  2NZ9A                834    0.615  88.679   0.458   0.938   0.007   0.055
E R  2NZ9A                835    0.372  85.188   0.965   0.970   0.001   0.030
B L  2NZ9A                836    0.056  10.180  -0.027   0.970   0.001   0.030
E K  2NZ9A                837    0.375  77.096   1.099   0.970   0.001   0.030
E D  2NZ9A                838    0.472  67.986  -0.014   0.970   0.001   0.030
B K  2NZ9A                839    0.193  39.680   1.004   0.970   0.001   0.030
B V  2NZ9A                840    0.068  10.390  -0.304   0.923   0.002   0.076
E N  2NZ9A                841    0.358  52.440   0.017   0.923   0.002   0.076
E N  2NZ9A                842    0.514  75.176  -0.232   0.782   0.003   0.216
B T  2NZ9A                843    0.191  26.519  -0.650   0.694   0.003   0.303
B L  2NZ9A                844    0.124  22.704  -0.932   0.430   0.016   0.555
E S  2NZ9A                845    0.543  63.651  -0.138   0.184   0.043   0.773
E T  2NZ9A                846    0.410  56.853  -1.129   0.113   0.043   0.844
E D  2NZ9A                847    0.456  65.710  -0.844   0.052   0.084   0.864
B I  2NZ9A                848    0.126  23.310   0.248   0.056   0.142   0.802
E P  2NZ9A                849    0.282  40.002  -0.337   0.056   0.142   0.802
B F  2NZ9A                850    0.157  31.430  -0.535   0.056   0.142   0.802
E Q  2NZ9A                851    0.544  97.141  -0.736   0.056   0.142   0.802
B L  2NZ9A                852    0.232  42.516  -1.029   0.113   0.087   0.800
B S  2NZ9A                853    0.231  27.120  -1.388   0.113   0.087   0.800
E K  2NZ9A                854    0.445  91.557   0.063   0.191   0.086   0.723
B Y  2NZ9A                855    0.205  43.873  -0.472   0.278   0.093   0.628
B V  2NZ9A                856    0.210  32.277  -0.383   0.268   0.043   0.689
E D  2NZ9A                857    0.378  54.484  -0.696   0.268   0.043   0.689
B N  2NZ9A                858    0.170  24.932  -0.312   0.622   0.015   0.363
E Q  2NZ9A                859    0.509  90.979   0.437   0.831   0.044   0.125
B R  2NZ9A                860    0.238  54.479   0.781   0.911   0.033   0.057
B L  2NZ9A                861    0.043   7.910   0.403   0.938   0.007   0.055
B L  2NZ9A                862    0.124  22.686   1.224   0.975   0.003   0.022
E S  2NZ9A                863    0.425  49.822  -0.052   0.970   0.001   0.030
B T  2NZ9A                864    0.078  10.860   0.877   0.970   0.001   0.030
B F  2NZ9A                865    0.022   4.395   1.011   0.970   0.001   0.030
B T  2NZ9A                866    0.236  32.775   0.667   0.970   0.001   0.030
E E  2NZ9A                867    0.528  92.207   1.110   0.970   0.001   0.030
B Y  2NZ9A                868    0.120  25.537   0.085   0.970   0.001   0.030
B I  2NZ9A                869    0.060  11.174   0.652   0.923   0.002   0.076
E K  2NZ9A                870    0.578 118.895   1.205   0.858   0.002   0.139
E N  2NZ9A                871    0.447  65.426   1.487   0.802   0.014   0.185
B I  2NZ9A                872    0.040   7.437  -0.485   0.717   0.014   0.269
E I  2NZ9A                873    0.267  49.377   0.493   0.430   0.016   0.555
E N  2NZ9A                874    0.695 101.792   0.223   0.257   0.016   0.727
E T  2NZ9A                875    0.494  68.462  -0.712   0.191   0.086   0.723
E S  2NZ9A                876    0.279  32.687   0.379   0.125   0.227   0.648
B I  2NZ9A                877    0.069  12.839  -0.665   0.074   0.484   0.442
B L  2NZ9A                878    0.125  22.924   0.481   0.023   0.655   0.322
B N  2NZ9A                879    0.170  24.873  -1.983   0.023   0.655   0.322
B L  2NZ9A                880    0.095  17.303  -0.156   0.023   0.655   0.322
E R  2NZ9A                881    0.353  80.814   0.113   0.022   0.552   0.426
B Y  2NZ9A                882    0.277  59.088  -0.407   0.021   0.279   0.699
E E  2NZ9A                883    0.682 119.093   0.248   0.018   0.047   0.935
E S  2NZ9A                884    0.718  84.185  -1.495   0.003   0.003   0.994
E N  2NZ9A                885    0.425  62.293  -0.657   0.005   0.015   0.979
E H  2NZ9A                886    0.327  59.409   1.102   0.004   0.514   0.481
B L  2NZ9A                887    0.063  11.554   0.193   0.002   0.816   0.182
B I  2NZ9A                888    0.137  25.364   0.233   0.011   0.918   0.071
B D  2NZ9A                889    0.136  19.526  -0.793   0.011   0.918   0.071
B L  2NZ9A                890    0.083  15.197  -0.674   0.023   0.655   0.322
B S  2NZ9A                891    0.164  19.244  -1.419   0.021   0.451   0.528
B R  2NZ9A                892    0.264  60.479  -1.421   0.005   0.336   0.660
B Y  2NZ9A                893    0.259  55.284  -0.423   0.005   0.262   0.733
B A  2NZ9A                894    0.319  35.176  -1.706   0.004   0.138   0.858
E S  2NZ9A                895    0.541  63.358  -1.983   0.004   0.138   0.858
E K  2NZ9A                896    0.419  86.127  -0.919   0.005   0.336   0.660
B I  2NZ9A                897    0.091  16.890  -0.656   0.003   0.718   0.279
E N  2NZ9A                898    0.355  52.001  -0.220   0.001   0.900   0.099
B I  2NZ9A                899    0.196  36.297  -0.497   0.001   0.900   0.099
B G  2NZ9A                900    0.274  21.580  -0.347   0.003   0.718   0.279
E S  2NZ9A                901    0.347  40.633  -1.531   0.005   0.336   0.660
E K  2NZ9A                902    0.439  90.343  -0.789   0.005   0.262   0.733
B V  2NZ9A                903    0.132  20.242  -0.537   0.004   0.616   0.381
B N  2NZ9A                904    0.285  41.768  -0.924   0.002   0.816   0.182
B F  2NZ9A                905    0.218  43.733  -0.526   0.021   0.756   0.223
B D  2NZ9A                906    0.212  30.564  -0.150   0.023   0.655   0.322
E P  2NZ9A                907    0.361  51.240  -1.560   0.022   0.359   0.619
B I  2NZ9A                908    0.219  40.571  -2.106   0.019   0.141   0.840
E D  2NZ9A                909    0.286  41.198  -0.874   0.019   0.141   0.840
E K  2NZ9A                910    0.352  72.365  -0.540   0.052   0.084   0.864
E N  2NZ9A                911    0.404  59.190  -1.926   0.056   0.142   0.802
E Q  2NZ9A                912    0.300  53.651  -0.382   0.022   0.359   0.619
B I  2NZ9A                913    0.097  18.037  -1.077   0.023   0.655   0.322
B Q  2NZ9A                914    0.239  42.721  -0.356   0.021   0.756   0.223
B L  2NZ9A                915    0.101  18.420  -0.963   0.021   0.756   0.223
B F  2NZ9A                916    0.195  39.056  -0.945   0.022   0.552   0.426
E N  2NZ9A                917    0.287  42.017  -1.290   0.021   0.279   0.699
E L  2NZ9A                918    0.394  72.086  -1.912   0.018   0.088   0.893
E E  2NZ9A                919    0.686 119.897  -0.631   0.018   0.047   0.935
E S  2NZ9A                920    0.450  52.752  -1.362   0.018   0.047   0.935
B S  2NZ9A                921    0.289  33.836  -1.196   0.004   0.138   0.858
E K  2NZ9A                922    0.490 100.855  -0.379   0.004   0.616   0.381
B I  2NZ9A                923    0.167  30.821  -0.131   0.001   0.900   0.099
E E  2NZ9A                924    0.329  57.476   0.757   0.001   0.959   0.040
B V  2NZ9A                925    0.056   8.592   0.422   0.001   0.959   0.040
E I  2NZ9A                926    0.357  66.008  -0.018   0.001   0.959   0.040
B L  2NZ9A                927    0.172  31.566   0.629   0.003   0.718   0.279
E K  2NZ9A                928    0.457  93.964  -0.918   0.020   0.205   0.775
E N  2NZ9A                929    0.439  64.328  -0.571   0.058   0.017   0.925
E A  2NZ9A                930    0.434  47.871  -0.541   0.064   0.216   0.721
B I  2NZ9A                931    0.128  23.717   0.389   0.021   0.756   0.223
E V  2NZ9A                932    0.256  39.301  -0.579   0.018   0.846   0.136
B Y  2NZ9A                933    0.194  41.436  -0.020   0.021   0.756   0.223
B N  2NZ9A                934    0.263  38.489  -1.429   0.022   0.359   0.619
E S  2NZ9A                935    0.378  44.255  -1.462   0.018   0.088   0.893
E M  2NZ9A                936    0.291  58.249  -0.899   0.019   0.141   0.840
E Y  2NZ9A                937    0.293  62.614  -1.120   0.019   0.141   0.840
E E  2NZ9A                938    0.397  69.303  -0.775   0.019   0.141   0.840
B N  2NZ9A                939    0.244  35.692  -1.369   0.004   0.197   0.799
B F  2NZ9A                940    0.045   8.991   0.357   0.003   0.718   0.279
B S  2NZ9A                941    0.055   6.505  -0.297   0.011   0.918   0.071
B T  2NZ9A                942    0.025   3.509   0.680   0.006   0.962   0.032
B S  2NZ9A                943    0.029   3.446   0.224   0.006   0.962   0.032
B F  2NZ9A                944    0.025   5.038   0.748   0.011   0.918   0.071
B W  2NZ9A                945    0.026   6.181   0.428   0.001   0.959   0.040
B I  2NZ9A                946    0.029   5.273   0.183   0.001   0.900   0.099
B R  2NZ9A                947    0.123  28.259   0.160   0.002   0.816   0.182
B I  2NZ9A                948    0.062  11.544  -0.074   0.021   0.451   0.528
B P  2NZ9A                949    0.259  36.724   0.039   0.020   0.205   0.775
E K  2NZ9A                950    0.476  97.810  -0.335   0.020   0.205   0.775
E Y  2NZ9A                951    0.301  64.388  -1.108   0.021   0.279   0.699
E F  2NZ9A                952    0.439  88.208  -1.396   0.020   0.205   0.775
E N  2NZ9A                953    0.421  61.605  -1.563   0.052   0.084   0.864
E S  2NZ9A                954    0.647  75.875  -2.486   0.053   0.043   0.903
E I  2NZ9A                955    0.455  84.231  -0.872   0.053   0.043   0.903
E S  2NZ9A                956    0.382  44.724  -1.929   0.053   0.043   0.903
E L  2NZ9A                957    0.285  52.165  -0.593   0.052   0.084   0.864
E N  2NZ9A                958    0.373  54.680  -1.174   0.018   0.088   0.893
E N  2NZ9A                959    0.416  60.932  -0.616   0.018   0.088   0.893
E E  2NZ9A                960    0.411  71.767  -0.241   0.020   0.205   0.775
B Y  2NZ9A                961    0.124  26.584   0.419   0.003   0.718   0.279
B T  2NZ9A                962    0.053   7.309   0.315   0.011   0.918   0.071
B I  2NZ9A                963    0.035   6.420   0.162   0.011   0.918   0.071
B I  2NZ9A                964    0.039   7.197  -0.282   0.011   0.918   0.071
B N  2NZ9A                965    0.102  14.918  -0.871   0.018   0.846   0.136
B C  2NZ9A                966    0.059   8.354  -0.233   0.023   0.655   0.322
B M  2NZ9A                967    0.243  48.664  -0.031   0.004   0.514   0.481
E E  2NZ9A                968    0.529  92.451  -0.575   0.022   0.359   0.619
E N  2NZ9A                969    0.421  61.693  -0.887   0.020   0.205   0.775
E N  2NZ9A                970    0.418  61.210  -1.850   0.018   0.088   0.893
B S  2NZ9A                971    0.245  28.691  -1.068   0.018   0.088   0.893
B G  2NZ9A                972    0.162  12.757  -2.256   0.004   0.197   0.799
B W  2NZ9A                973    0.104  24.964   0.007   0.004   0.616   0.381
B K  2NZ9A                974    0.275  56.506   0.396   0.002   0.816   0.182
B V  2NZ9A                975    0.081  12.419   0.468   0.001   0.900   0.099
B S  2NZ9A                976    0.129  15.154  -0.580   0.001   0.959   0.040
B L  2NZ9A                977    0.063  11.517  -0.163   0.001   0.900   0.099
E N  2NZ9A                978    0.339  49.644   0.615   0.004   0.616   0.381
E Y  2NZ9A                979    0.535 114.223  -1.536   0.004   0.138   0.858
B G  2NZ9A                980    0.213  16.747  -0.934   0.004   0.138   0.858
B E  2NZ9A                981    0.209  36.425  -0.530   0.004   0.420   0.576
B I  2NZ9A                982    0.052   9.601   0.696   0.001   0.900   0.099
B I  2NZ9A                983    0.078  14.338   0.600   0.001   0.959   0.040
B W  2NZ9A                984    0.045  10.726   0.821   0.001   0.959   0.040
B T  2NZ9A                985    0.126  17.490  -0.220   0.001   0.959   0.040
B L  2NZ9A                986    0.063  11.535   0.886   0.001   0.900   0.099
E Q  2NZ9A                987    0.298  53.241   1.292   0.001   0.900   0.099
B D  2NZ9A                988    0.272  39.138  -0.960   0.004   0.514   0.481
E T  2NZ9A                989    0.502  69.572  -1.549   0.018   0.088   0.893
E Q  2NZ9A                990    0.603 107.607  -1.649   0.018   0.047   0.935
E E  2NZ9A                991    0.621 108.489  -1.390   0.018   0.047   0.935
E I  2NZ9A                992    0.476  87.986  -0.570   0.019   0.141   0.840
E K  2NZ9A                993    0.503 103.447  -0.052   0.022   0.552   0.426
E Q  2NZ9A                994    0.261  46.543   0.897   0.021   0.756   0.223
E R  2NZ9A                995    0.378  86.470   0.358   0.018   0.846   0.136
B V  2NZ9A                996    0.068  10.452   0.539   0.018   0.846   0.136
B V  2NZ9A                997    0.121  18.598  -0.769   0.018   0.846   0.136
B F  2NZ9A                998    0.158  31.650  -0.059   0.023   0.655   0.322
E K  2NZ9A                999    0.437  89.891  -0.926   0.069   0.386   0.545
B Y  2NZ9A               1000    0.215  46.031  -0.041   0.118   0.150   0.732
E S  2NZ9A               1001    0.418  49.036  -1.104   0.113   0.087   0.800
B Q  2NZ9A               1002    0.285  50.812  -0.860   0.199   0.152   0.649
E M  2NZ9A               1003    0.367  73.457  -0.903   0.199   0.152   0.649
E I  2NZ9A               1004    0.478  88.412  -1.287   0.191   0.086   0.723
E N  2NZ9A               1005    0.408  59.775  -1.566   0.113   0.087   0.800
B I  2NZ9A               1006    0.165  30.580  -1.139   0.191   0.086   0.723
E S  2NZ9A               1007    0.333  38.981  -1.352   0.113   0.043   0.844
E D  2NZ9A               1008    0.434  62.568  -1.541   0.113   0.043   0.844
B Y  2NZ9A               1009    0.186  39.684  -0.750   0.052   0.084   0.864
B I  2NZ9A               1010    0.284  52.485  -1.079   0.018   0.047   0.935
B N  2NZ9A               1011    0.284  41.534  -1.350   0.018   0.088   0.893
E R  2NZ9A               1012    0.228  52.281   0.967   0.005   0.336   0.660
B W  2NZ9A               1013    0.040   9.740   0.179   0.002   0.816   0.182
B I  2NZ9A               1014    0.055  10.249   0.957   0.001   0.959   0.040
B F  2NZ9A               1015    0.058  11.721   0.584   0.001   0.959   0.040
B V  2NZ9A               1016    0.021   3.258   1.136   0.001   0.959   0.040
B T  2NZ9A               1017    0.043   5.895   0.499   0.001   0.959   0.040
B I  2NZ9A               1018    0.030   5.531   0.497   0.001   0.959   0.040
B T  2NZ9A               1019    0.071   9.875  -0.230   0.002   0.816   0.182
B N  2NZ9A               1020    0.152  22.224  -0.257   0.004   0.514   0.481
B N  2NZ9A               1021    0.246  36.073  -0.532   0.020   0.205   0.775
E R  2NZ9A               1022    0.337  77.173  -0.321   0.019   0.141   0.840
B L  2NZ9A               1023    0.268  49.126  -1.355   0.019   0.141   0.840
E N  2NZ9A               1024    0.459  67.271  -0.664   0.018   0.088   0.893
E N  2NZ9A               1025    0.347  50.772  -0.895   0.020   0.205   0.775
B S  2NZ9A               1026    0.105  12.247  -0.530   0.023   0.655   0.322
B K  2NZ9A               1027    0.177  36.429   0.631   0.018   0.846   0.136
B I  2NZ9A               1028    0.046   8.455   0.885   0.011   0.918   0.071
B Y  2NZ9A               1029    0.098  20.964   0.318   0.018   0.846   0.136
B I  2NZ9A               1030    0.092  17.020   0.079   0.021   0.756   0.223
E N  2NZ9A               1031    0.307  44.886  -0.445   0.021   0.279   0.699
E G  2NZ9A               1032    0.362  28.497  -1.624   0.019   0.141   0.840
E R  2NZ9A               1033    0.492 112.554   0.265   0.022   0.552   0.426
B L  2NZ9A               1034    0.177  32.354  -0.259   0.023   0.655   0.322
E I  2NZ9A               1035    0.390  72.113   0.190   0.022   0.552   0.426
E D  2NZ9A               1036    0.457  65.897  -0.506   0.021   0.451   0.528
E Q  2NZ9A               1037    0.578 103.302   0.236   0.069   0.386   0.545
E K  2NZ9A               1038    0.365  75.163   0.817   0.074   0.484   0.442
E P  2NZ9A               1039    0.492  69.801   1.124   0.074   0.484   0.442
B I  2NZ9A               1040    0.048   8.880   0.373   0.252   0.423   0.325
E S  2NZ9A               1041    0.432  50.584   1.341   0.216   0.235   0.548
E N  2NZ9A               1042    0.474  69.364  -0.131   0.307   0.165   0.527
B L  2NZ9A               1043    0.111  20.361   0.076   0.278   0.093   0.628
B G  2NZ9A               1044    0.248  19.510  -1.343   0.199   0.152   0.649
E N  2NZ9A               1045    0.428  62.674  -0.127   0.118   0.150   0.732
B I  2NZ9A               1046    0.079  14.634   0.346   0.135   0.317   0.548
B H  2NZ9A               1047    0.271  49.313   0.240   0.148   0.418   0.435
B A  2NZ9A               1048    0.170  18.734  -0.679   0.135   0.317   0.548
B S  2NZ9A               1049    0.286  33.531  -0.797   0.052   0.084   0.864
E N  2NZ9A               1050    0.347  50.742  -0.933   0.053   0.043   0.903
B N  2NZ9A               1051    0.261  38.137  -0.172   0.064   0.216   0.721
B I  2NZ9A               1052    0.056  10.416  -0.072   0.079   0.592   0.329
B M  2NZ9A               1053    0.167  33.477  -0.274   0.087   0.683   0.230
B F  2NZ9A               1054    0.137  27.476   0.162   0.087   0.683   0.230
E K  2NZ9A               1055    0.301  61.977  -0.212   0.087   0.683   0.230
B L  2NZ9A               1056    0.156  28.545  -0.997   0.021   0.451   0.528
B D  2NZ9A               1057    0.316  45.579  -1.609   0.022   0.359   0.619
E G  2NZ9A               1058    0.356  28.049  -0.897   0.021   0.279   0.699
B C  2NZ9A               1059    0.068   9.589  -0.553   0.021   0.279   0.699
E R  2NZ9A               1060    0.386  88.440   0.022   0.020   0.205   0.775
E D  2NZ9A               1061    0.401  57.726  -1.555   0.052   0.084   0.864
E T  2NZ9A               1062    0.546  75.786  -0.635   0.053   0.043   0.903
E H  2NZ9A               1063    0.339  61.591  -1.420   0.053   0.043   0.903
E R  2NZ9A               1064    0.287  65.838   0.165   0.064   0.216   0.721
B Y  2NZ9A               1065    0.179  38.188  -0.464   0.079   0.592   0.329
B I  2NZ9A               1066    0.053   9.805   0.470   0.085   0.775   0.140
B W  2NZ9A               1067    0.081  19.384  -0.275   0.072   0.855   0.073
B I  2NZ9A               1068    0.031   5.790   0.090   0.172   0.690   0.138
E K  2NZ9A               1069    0.277  56.897   0.280   0.252   0.423   0.325
B Y  2NZ9A               1070    0.193  41.159  -0.528   0.352   0.332   0.316
B F  2NZ9A               1071    0.041   8.148   0.565   0.373   0.401   0.226
E N  2NZ9A               1072    0.296  43.334   0.673   0.373   0.401   0.226
B L  2NZ9A               1073    0.061  11.206   0.004   0.478   0.309   0.214
B F  2NZ9A               1074    0.088  17.682   0.041   0.428   0.171   0.402
B D  2NZ9A               1075    0.251  36.155  -0.036   0.278   0.093   0.628
E K  2NZ9A               1076    0.438  90.055   1.280   0.354   0.048   0.598
E E  2NZ9A               1077    0.496  86.581  -0.575   0.354   0.048   0.598
B L  2NZ9A               1078    0.116  21.221   0.450   0.115   0.016   0.868
E N  2NZ9A               1079    0.458  66.993   0.720   0.018   0.019   0.964
E E  2NZ9A               1080    0.445  77.811   0.693   0.923   0.002   0.076
E K  2NZ9A               1081    0.688 141.522   1.280   0.923   0.002   0.076
E E  2NZ9A               1082    0.255  44.479   0.674   0.923   0.002   0.076
B I  2NZ9A               1083    0.030   5.494   0.141   0.970   0.001   0.030
E K  2NZ9A               1084    0.330  67.984   1.184   0.970   0.001   0.030
E D  2NZ9A               1085    0.507  73.044   0.871   0.923   0.002   0.076
B L  2NZ9A               1086    0.092  16.772   0.631   0.858   0.002   0.139
B Y  2NZ9A               1087    0.101  21.669  -0.018   0.802   0.014   0.185
E D  2NZ9A               1088    0.429  61.804  -0.647   0.717   0.014   0.269
E N  2NZ9A               1089    0.384  56.232  -1.144   0.522   0.016   0.462
B Q  2NZ9A               1090    0.256  45.632  -0.699   0.339   0.016   0.645
E S  2NZ9A               1091    0.336  39.379  -1.175   0.181   0.016   0.803
E N  2NZ9A               1092    0.411  60.097  -1.543   0.115   0.016   0.868
B S  2NZ9A               1093    0.315  36.965  -1.222   0.113   0.043   0.844
E G  2NZ9A               1094    0.327  25.751  -1.406   0.118   0.150   0.732
B I  2NZ9A               1095    0.164  30.321   0.130   0.135   0.317   0.548
B L  2NZ9A               1096    0.076  13.952  -0.371   0.159   0.516   0.325
E K  2NZ9A               1097    0.340  69.897  -0.036   0.148   0.418   0.435
B D  2NZ9A               1098    0.294  42.322  -1.741   0.125   0.227   0.648
B F  2NZ9A               1099    0.256  51.439  -1.221   0.052   0.084   0.864
E W  2NZ9A               1100    0.342  82.275  -1.402   0.052   0.084   0.864
E G  2NZ9A               1101    0.481  37.878  -1.566   0.053   0.043   0.903
E D  2NZ9A               1102    0.475  68.448  -1.167   0.053   0.043   0.903
E Y  2NZ9A               1103    0.351  75.009   0.488   0.118   0.150   0.732
B L  2NZ9A               1104    0.091  16.699  -0.274   0.066   0.296   0.638
E Q  2NZ9A               1105    0.349  62.349  -0.155   0.069   0.386   0.545
B Y  2NZ9A               1106    0.263  56.118  -0.397   0.069   0.386   0.545
E D  2NZ9A               1107    0.430  62.035  -0.309   0.064   0.216   0.721
E K  2NZ9A               1108    0.408  83.843   0.119   0.064   0.216   0.721
E P  2NZ9A               1109    0.454  64.352   0.813   0.074   0.484   0.442
B Y  2NZ9A               1110    0.119  25.494   0.710   0.023   0.655   0.322
B Y  2NZ9A               1111    0.155  33.145   0.252   0.087   0.683   0.230
B M  2NZ9A               1112    0.072  14.347  -0.111   0.087   0.683   0.230
B L  2NZ9A               1113    0.075  13.678   0.660   0.079   0.592   0.329
B N  2NZ9A               1114    0.152  22.311  -0.301   0.079   0.592   0.329
B L  2NZ9A               1115    0.107  19.555   0.283   0.074   0.484   0.442
E Y  2NZ9A               1116    0.291  62.272  -1.253   0.069   0.386   0.545
E D  2NZ9A               1117    0.288  41.472   0.308   0.019   0.141   0.840
E P  2NZ9A               1118    0.408  57.966  -0.843   0.018   0.047   0.935
E N  2NZ9A               1119    0.689 100.811  -1.496   0.018   0.047   0.935
E K  2NZ9A               1120    0.349  71.872   0.409   0.056   0.142   0.802
B Y  2NZ9A               1121    0.219  46.715  -0.564   0.069   0.386   0.545
B V  2NZ9A               1122    0.067  10.359   0.044   0.087   0.683   0.230
E D  2NZ9A               1123    0.284  40.968   0.202   0.085   0.775   0.140
E V  2NZ9A               1124    0.306  47.032   0.210   0.087   0.683   0.230
B N  2NZ9A               1125    0.287  41.944  -0.639   0.079   0.592   0.329
E N  2NZ9A               1126    0.399  58.399  -0.856   0.066   0.296   0.638
E V  2NZ9A               1127    0.584  89.715  -1.180   0.064   0.216   0.721
E G  2NZ9A               1128    0.530  41.750  -1.241   0.056   0.142   0.802
E I  2NZ9A               1129    0.322  59.551  -1.234   0.064   0.216   0.721
E R  2NZ9A               1130    0.431  98.745   0.296   0.079   0.592   0.329
B G  2NZ9A               1131    0.125   9.845  -1.422   0.021   0.756   0.223
B Y  2NZ9A               1132    0.149  31.863   0.081   0.018   0.846   0.136
B M  2NZ9A               1133    0.046   9.125  -0.318   0.018   0.846   0.136
B Y  2NZ9A               1134    0.197  42.013   0.179   0.021   0.756   0.223
B L  2NZ9A               1135    0.082  15.014  -1.035   0.021   0.756   0.223
E K  2NZ9A               1136    0.288  59.262  -0.523   0.021   0.451   0.528
B G  2NZ9A               1137    0.256  20.155  -0.819   0.064   0.216   0.721
E P  2NZ9A               1138    0.390  55.369  -1.112   0.056   0.142   0.802
E R  2NZ9A               1139    0.491 112.439  -1.381   0.052   0.084   0.864
E G  2NZ9A               1140    0.410  32.228  -1.921   0.056   0.142   0.802
B S  2NZ9A               1141    0.212  24.893  -1.082   0.135   0.317   0.548
E V  2NZ9A               1142    0.338  51.904  -1.361   0.066   0.296   0.638
B M  2NZ9A               1143    0.244  48.924  -1.679   0.135   0.317   0.548
E T  2NZ9A               1144    0.409  56.742  -0.529   0.118   0.150   0.732
B T  2NZ9A               1145    0.181  25.063  -2.753   0.118   0.150   0.732
B N  2NZ9A               1146    0.211  30.876  -0.871   0.148   0.418   0.435
B I  2NZ9A               1147    0.172  31.820  -0.424   0.169   0.612   0.219
B Y  2NZ9A               1148    0.114  24.405  -0.926   0.159   0.516   0.325
B L  2NZ9A               1149    0.185  33.819  -0.754   0.148   0.418   0.435
B N  2NZ9A               1150    0.251  36.702  -1.764   0.216   0.235   0.548
B S  2NZ9A               1151    0.310  36.355  -1.709   0.118   0.150   0.732
E S  2NZ9A               1152    0.447  52.377  -0.912   0.113   0.087   0.800
B L  2NZ9A               1153    0.178  32.555  -1.461   0.191   0.086   0.723
B Y  2NZ9A               1154    0.200  42.804  -1.728   0.191   0.086   0.723
E R  2NZ9A               1155    0.380  86.951  -0.353   0.118   0.150   0.732
B G  2NZ9A               1156    0.254  19.990  -1.580   0.125   0.227   0.648
B T  2NZ9A               1157    0.093  12.857   0.230   0.074   0.484   0.442
E K  2NZ9A               1158    0.295  60.723   0.708   0.072   0.855   0.073
B F  2NZ9A               1159    0.096  19.187   0.425   0.072   0.855   0.073
B I  2NZ9A               1160    0.094  17.298   0.127   0.072   0.855   0.073
B I  2NZ9A               1161    0.070  13.042  -0.193   0.072   0.855   0.073
E K  2NZ9A               1162    0.316  65.063   0.570   0.085   0.775   0.140
E K  2NZ9A               1163    0.271  55.662   0.081   0.087   0.683   0.230
B Y  2NZ9A               1164    0.150  32.098  -0.141   0.069   0.386   0.545
E A  2NZ9A               1165    0.454  50.009  -0.446   0.019   0.141   0.840
E S  2NZ9A               1166    0.518  60.710  -1.475   0.018   0.088   0.893
E G  2NZ9A               1167    0.637  50.108  -1.313   0.018   0.047   0.935
E N  2NZ9A               1168    0.559  81.852  -0.860   0.053   0.043   0.903
E K  2NZ9A               1169    0.505 103.940  -0.810   0.113   0.043   0.844
E D  2NZ9A               1170    0.392  56.545   0.181   0.184   0.043   0.773
E N  2NZ9A               1171    0.382  55.983  -0.139   0.199   0.152   0.649
B I  2NZ9A               1172    0.242  44.696   1.163   0.079   0.592   0.329
B V  2NZ9A               1173    0.058   8.915  -0.090   0.085   0.775   0.140
E R  2NZ9A               1174    0.315  72.158   0.775   0.023   0.655   0.322
E N  2NZ9A               1175    0.546  80.008   0.000   0.022   0.359   0.619
E N  2NZ9A               1176    0.379  55.515  -0.391   0.004   0.085   0.910
B D  2NZ9A               1177    0.253  36.443  -0.965   0.004   0.197   0.799
B R  2NZ9A               1178    0.168  38.541   0.647   0.001   0.900   0.099
B V  2NZ9A               1179    0.020   3.028   1.165   0.001   0.959   0.040
B Y  2NZ9A               1180    0.060  12.758   1.283   0.001   0.959   0.040
B I  2NZ9A               1181    0.019   3.478   1.446   0.001   0.959   0.040
B N  2NZ9A               1182    0.063   9.223   0.198   0.001   0.959   0.040
B V  2NZ9A               1183    0.035   5.395   0.289   0.001   0.900   0.099
B V  2NZ9A               1184    0.140  21.487  -0.198   0.002   0.816   0.182
E V  2NZ9A               1185    0.278  42.729  -0.112   0.004   0.616   0.381
E K  2NZ9A               1186    0.731 150.387  -0.595   0.004   0.085   0.910
E N  2NZ9A               1187    0.619  90.592  -0.321   0.018   0.047   0.935
E K  2NZ9A               1188    0.549 112.970   0.426   0.004   0.514   0.481
E E  2NZ9A               1189    0.436  76.152   1.310   0.002   0.816   0.182
B Y  2NZ9A               1190    0.126  26.926   0.341   0.001   0.900   0.099
B R  2NZ9A               1191    0.240  54.937   0.439   0.001   0.900   0.099
B L  2NZ9A               1192    0.075  13.824   0.002   0.001   0.900   0.099
B A  2NZ9A               1193    0.147  16.177  -0.408   0.018   0.846   0.136
B T  2NZ9A               1194    0.088  12.164  -0.595   0.018   0.846   0.136
B N  2NZ9A               1195    0.270  39.587  -0.472   0.023   0.655   0.322
B A  2NZ9A               1196    0.240  26.415  -0.739   0.005   0.336   0.660
E S  2NZ9A               1197    0.545  63.921  -1.544   0.020   0.205   0.775
E Q  2NZ9A               1198    0.472  84.281  -1.712   0.018   0.088   0.893
E A  2NZ9A               1199    0.533  58.737  -0.255   0.052   0.084   0.864
E G  2NZ9A               1200    0.594  46.771  -0.434   0.113   0.087   0.800
E V  2NZ9A               1201    0.412  63.386  -0.535   0.118   0.150   0.732
E E  2NZ9A               1202    0.438  76.484  -0.797   0.135   0.317   0.548
E K  2NZ9A               1203    0.464  95.486   0.323   0.074   0.484   0.442
E I  2NZ9A               1204    0.333  61.624   0.934   0.018   0.846   0.136
B L  2NZ9A               1205    0.123  22.466   0.300   0.011   0.918   0.071
E S  2NZ9A               1206    0.248  29.112   0.578   0.011   0.918   0.071
B A  2NZ9A               1207    0.147  16.243  -0.468   0.011   0.918   0.071
B L  2NZ9A               1208    0.093  17.010  -0.011   0.018   0.846   0.136
E E  2NZ9A               1209    0.368  64.237  -0.357   0.021   0.756   0.223
B I  2NZ9A               1210    0.098  18.112  -1.064   0.004   0.420   0.576
E P  2NZ9A               1211    0.545  77.265  -0.954   0.004   0.138   0.858
E D  2NZ9A               1212    0.471  67.828  -0.900   0.005   0.045   0.951
E V  2NZ9A               1213    0.385  59.144  -1.037   0.018   0.019   0.964
E G  2NZ9A               1214    0.658  51.808  -1.793   0.018   0.019   0.964
E N  2NZ9A               1215    0.482  70.609  -0.238   0.053   0.043   0.903
B L  2NZ9A               1216    0.119  21.752  -1.544   0.056   0.142   0.802
E S  2NZ9A               1217    0.317  37.117  -0.971   0.021   0.279   0.699
E Q  2NZ9A               1218    0.361  64.510  -0.082   0.022   0.552   0.426
B V  2NZ9A               1219    0.120  18.505   0.037   0.018   0.846   0.136
B V  2NZ9A               1220    0.042   6.502  -0.256   0.001   0.959   0.040
B V  2NZ9A               1221    0.103  15.846  -0.642   0.001   0.959   0.040
B M  2NZ9A               1222    0.062  12.486  -0.161   0.001   0.959   0.040
E K  2NZ9A               1223    0.305  62.739   0.211   0.001   0.900   0.099
B S  2NZ9A               1224    0.192  22.502  -1.774   0.002   0.816   0.182
E K  2NZ9A               1225    0.379  77.981  -0.163   0.003   0.718   0.279
E N  2NZ9A               1226    0.416  60.976  -1.376   0.004   0.420   0.576
E D  2NZ9A               1227    0.480  69.154  -1.624   0.020   0.205   0.775
E Q  2NZ9A               1228    0.586 104.660  -1.568   0.020   0.205   0.775
E G  2NZ9A               1229    0.504  39.673  -1.333   0.020   0.205   0.775
B I  2NZ9A               1230    0.269  49.728  -1.103   0.021   0.279   0.699
B T  2NZ9A               1231    0.291  40.306  -1.361   0.021   0.451   0.528
B N  2NZ9A               1232    0.180  26.381  -0.780   0.022   0.552   0.426
E K  2NZ9A               1233    0.273  56.074   0.069   0.021   0.756   0.223
B C  2NZ9A               1234    0.022   3.117   0.592   0.018   0.846   0.136
B K  2NZ9A               1235    0.163  33.591   0.138   0.011   0.918   0.071
B M  2NZ9A               1236    0.054  10.725   0.048   0.011   0.918   0.071
B N  2NZ9A               1237    0.195  28.548   0.586   0.011   0.918   0.071
B L  2NZ9A               1238    0.048   8.752   0.066   0.018   0.846   0.136
E Q  2NZ9A               1239    0.290  51.740   0.467   0.023   0.655   0.322
E D  2NZ9A               1240    0.357  51.501  -0.281   0.022   0.359   0.619
E N  2NZ9A               1241    0.673  98.586  -1.317   0.018   0.088   0.893
E N  2NZ9A               1242    0.639  93.491  -0.636   0.018   0.019   0.964
E G  2NZ9A               1243    0.514  40.452  -1.290   0.018   0.019   0.964
E N  2NZ9A               1244    0.504  73.756  -0.725   0.005   0.045   0.951
E D  2NZ9A               1245    0.339  48.821   0.069   0.022   0.359   0.619
B I  2NZ9A               1246    0.059  11.007   0.342   0.021   0.756   0.223
B G  2NZ9A               1247    0.077   6.084  -1.317   0.018   0.846   0.136
B F  2NZ9A               1248    0.064  12.765  -0.674   0.021   0.756   0.223
B I  2NZ9A               1249    0.039   7.252  -0.459   0.021   0.756   0.223
B G  2NZ9A               1250    0.044   3.431  -1.464   0.023   0.655   0.322
B F  2NZ9A               1251    0.061  12.162  -0.824   0.023   0.655   0.322
B H  2NZ9A               1252    0.147  26.739  -0.212   0.023   0.655   0.322
E Q  2NZ9A               1253    0.245  43.793  -0.623   0.023   0.655   0.322
B F  2NZ9A               1254    0.129  25.910  -0.401   0.022   0.552   0.426
E N  2NZ9A               1255    0.374  54.783  -0.724   0.022   0.359   0.619
E N  2NZ9A               1256    0.457  66.919  -1.115   0.056   0.142   0.802
E I  2NZ9A               1257    0.422  78.144  -1.708   0.018   0.047   0.935
E A  2NZ9A               1258    0.556  61.315  -2.373   0.018   0.019   0.964
E K  2NZ9A               1259    0.313  64.322  -0.036   0.064   0.216   0.721
B L  2NZ9A               1260    0.080  14.648   0.686   0.021   0.756   0.223
B V  2NZ9A               1261    0.068  10.452   0.181   0.018   0.846   0.136
B A  2NZ9A               1262    0.135  14.855   0.115   0.018   0.846   0.136
B S  2NZ9A               1263    0.077   9.048  -0.864   0.087   0.683   0.230
B N  2NZ9A               1264    0.213  31.125  -0.255   0.079   0.592   0.329
B W  2NZ9A               1265    0.136  32.684  -0.050   0.159   0.516   0.325
B Y  2NZ9A               1266    0.118  25.152  -0.390   0.252   0.423   0.325
B N  2NZ9A               1267    0.112  16.455   0.323   0.428   0.171   0.402
E R  2NZ9A               1268    0.417  95.401   0.963   0.502   0.102   0.396
B Q  2NZ9A               1269    0.288  51.401   0.436   0.502   0.102   0.396
B I  2NZ9A               1270    0.069  12.747   0.104   0.538   0.173   0.289
E E  2NZ9A               1271    0.467  81.498   0.651   0.428   0.171   0.402
E R  2NZ9A               1272    0.610 139.759  -0.063   0.278   0.093   0.628
E S  2NZ9A               1273    0.558  65.421  -1.262   0.184   0.043   0.773
E S  2NZ9A               1274    0.488  57.158  -1.022   0.058   0.017   0.925
E R  2NZ9A               1275    0.603 138.018  -0.742   0.053   0.043   0.903
E T  2NZ9A               1276    0.437  60.667  -1.343   0.018   0.088   0.893
B L  2NZ9A               1277    0.229  41.875  -0.930   0.018   0.088   0.893
B G  2NZ9A               1278    0.162  12.726  -2.452   0.004   0.085   0.910
B C  2NZ9A               1279    0.088  12.341  -0.288   0.004   0.197   0.799
B S  2NZ9A               1280    0.182  21.319  -0.648   0.004   0.514   0.481
B W  2NZ9A               1281    0.053  12.819  -0.239   0.002   0.816   0.182
B E  2NZ9A               1282    0.168  29.419  -1.188   0.001   0.900   0.099
B F  2NZ9A               1283    0.093  18.705  -0.311   0.001   0.900   0.099
B I  2NZ9A               1284    0.060  11.026   1.034   0.021   0.756   0.223
B P  2NZ9A               1285    0.103  14.616   0.377   0.022   0.552   0.426
E V  2NZ9A               1286    0.353  54.241   0.566   0.019   0.141   0.840
E D  2NZ9A               1287    0.591  85.134  -1.295   0.018   0.088   0.893
E D  2NZ9A               1288    0.496  71.488  -0.039   0.058   0.017   0.925
E G  2NZ9A               1289    0.418  32.912  -1.587   0.053   0.043   0.903
B W  2NZ9A               1290    0.167  40.091  -0.274   0.019   0.141   0.840
E G  2NZ9A               1291    0.533  41.931   0.165   0.021   0.279   0.699
E E  2NZ9A               1292    0.484  84.625  -0.848   0.021   0.279   0.699
E R  2NZ9A               1293    0.519 118.874   0.295   0.020   0.205   0.775
E P  2NZ9A               1294    0.547  77.563  -1.053   0.018   0.088   0.893
E L  2NZ9A               1295    0.779 142.653  -0.777   0.003   0.003   0.994

Explain the output.  Go back.
```

---
